# Rational Grain Boundary Segregation Enables Long‐Term Thermally Stable, Catalytically Active, and CO‐Tolerant Nanograined Metals

**DOI:** 10.1002/advs.202512730

**Published:** 2025-09-20

**Authors:** Xin Geng, Xiaolong Lu, Zhenyu Wang, Baptiste Gault

**Affiliations:** ^1^ Max Planck Institute for Sustainable Materials Max‐Planck‐Straße 1 40237 Düsseldorf Germany; ^2^ School of Intelligent Manufacturing Ecosystem Xi'an Jiaotong‐Liverpool University Suzhou 215123 China; ^3^ Department of Materials Royal School of Mines Imperial College London London SW7 2AZ UK

**Keywords:** anti‐CO poisoning, atom probe tomography, GB engineering, GB segregation, thermal stability

## Abstract

Grain boundaries (GBs) impart nanocrystalline metals with unique functional properties but are often plagued by poor thermal stability. While solute segregation is commonly used to stabilize GBs, stability alone is insufficient—effective strategies must also preserve, or ideally enhance, functional activity, a critical aspect largely overlooked. Here, it is shown that GB segregation is not universally beneficial: the same segregant can either stabilize or destabilize GBs depending on its local concentration and spatial distribution. Using boron‐segregated platinum as an example, a concentration‐dependent duality is uncovered: at sub‐equilibrium levels, boron strengthens GB cohesion and preserves structure during prolonged annealing; when oversaturated, boron clusters near GBs, generating repulsive interactions that trigger decohesion. In extreme cases, segregation can render GBs less stable than those without segregants. To address this, a three‐step framework is established for rational GB design: 1) theoretical selection of segregants with strong GB affinity and minimal impact on catalytic energetics, 2) thermodynamic determination of segregation limits, and 3) kinetic trapping during nanoparticle attachment for targeted GB incorporation. Guided by this approach, thermally stable, GB‐rich platinum nanograins that retain high catalytic activity and exhibit enhanced carbon monoxide tolerance are fabricated. This work provides a blueprint for engineering robust, high‐performance nanocrystalline materials.

## Graphical abstract

Grain boundaries endow nanocrystalline metals with unique functions but suffer thermal instability. In boron‐segregated platinum, stability depends critically on segregation level—sub‐equilibrium concentrations strengthen boundaries, whereas oversaturation induces decohesion. A three‐step design framework enables fabrication of thermally stable, catalytically active Pt nanograins with enhanced CO tolerance, offering a blueprint for robust nanocrystalline materials.

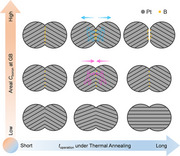

## 1 Introduction

The refinement of grain size to the nanometer regime has revolutionized the design of metallic materials, offering access to unprecedented combinations of mechanical strength, chemical reactivity, and functional performance.^[^ ^1^, ^2^, ^3^, ^4^, ^5^, ^6^, ^7^, ^8^ ^]^ These benefits arise from their high density of grain boundaries (GBs),^[^ ^9^ ^]^ which serve not only as barriers to dislocation motion^[^ ^10^, ^11^, ^12^ ^]^ but also as active sites for catalysis.^[^ ^13^, ^14^, ^15^ ^]^ However, the very features that confer such advantages also give rise to profound limitations. The high density of GBs renders nanograined materials inherently metastable, with a strong thermodynamic driving force for grain coarsening.^[^ ^16^, ^17^ ^]^ This process, driven primarily by GB migration,^[^ ^18^, ^19^ ^]^ erodes the nanostructure over time, often reversing the beneficial properties imparted by grain refinement. The challenge becomes increasingly acute as the grain size decreases: the so‐called instability temperature—below which the microstructure remains stable—drops sharply at the nanoscale.^[^ ^1^, ^20^, ^21^, ^22^ ^]^ In extreme cases, such as nanocrystalline copper,^[^ ^23^ ^]^ structural degradation can begin at ambient conditions, while in platinum,^[^ ^19^ ^]^ grain growth may initiate at temperatures as low as 200 °C. These observations underscore a fundamental limitation in the thermal robustness of nanograined metals and highlight the urgent need for stabilization strategies that are both effective and scalable.

At the core of this instability lies the complex interplay between GB structure,^[^ ^24^, ^25^, ^26^, ^27^, ^28^ ^]^ chemistry,^[^ ^29^ ^]^ and external stimuli.^[^ ^24^, ^25^, ^26^, ^30^ ^]^ Elevated temperatures^[^ ^24^, ^25^, ^26^ ^]^ or intense electric fields^[^ ^30^ ^]^ can significantly enhance atomic mobility, particularly along GBs, where chemical heterogeneity and structural disorder promote rapid diffusion. Under these conditions, atoms—especially those misfitting or energetically unfavorable—can redistribute or migrate, triggering boundary movement and grain growth. Stabilizing nanocrystalline metals thus requires not only slowing this migration but doing so in a manner that does not compromise the functional roles of GBs.

Efforts to counteract GB instability have largely followed two paradigms: thermodynamic stabilization,^[^ ^31^, ^32^ ^]^ where solute segregation lowers GB energy, and kinetic stabilization,^[^ ^29^ ^]^ where boundary motion is impeded by pinning mechanisms or chemical barriers. While both approaches have shown promise, their effectiveness is highly system‐dependent.^[^ ^33^, ^34^, ^35^ ^]^ Importantly, not all segregants are beneficial—certain elements, including hydrogen,^[^ ^36^ ^]^ sulfur,^[^ ^37^, ^38^ ^]^ and phosphorus,^[^ ^37^ ^]^ may reduce GB energy but at the cost of cohesion, embrittling the interface and degrading mechanical reliability.^[^ ^36^ ^]^ Moreover, in functionally active materials such as catalysts, GB stabilization must meet an additional criterion: the preservation—or enhancement—of activity. If the introduction of a segregant compromises the catalytic efficacy of a GB, then its stabilizing effect is rendered moot from an application standpoint. This dual requirement—structural stability and functional retention—represents a central, unsolved problem in the design of nanograined materials. Addressing it demands a shift from empirical trial‐and‐error approaches toward predictive, mechanism‐based strategies that consider not only the energetics of segregation but also its impact on GB‐mediated functionality.

Compounding the challenge is the difficulty of characterizing segregants at the relevant length scales. Many effective segregants are present in low concentrations or possess low atomic numbers, making them difficult to detect with conventional techniques such as X‐ray photoelectron spectroscopy or energy‐dispersive X‐ray analysis.^[^ ^39^, ^40^ ^]^ These limitations hinder the ability to establish clear structure—composition–property relationships at GBs. Recent advances in atomic‐scale characterization, particularly atom probe tomography,^[^ ^41^, ^42^ ^]^ now offer a pathway forward. By providing 3D chemical maps with near‐atomic resolution, these tools make it possible to directly observe and quantify segregation phenomena within individual GBs—offering the data necessary to validate models and refine design principles.

Here, we present a rational segregation‐enabled strategy to engineer GB‐connected platinum (Pt) nanoparticles (NPs) that simultaneously exhibit high catalytic activity, strong CO tolerance, and exceptional thermal stability. By assembling ≈2 nm Pt NPs into continuous architectures enriched with GBs, we construct GB‐rich networks that demonstrate enhanced thermal catalytic hydrogen oxidation reaction (TCHOR) activity and robust CO tolerance. However, the high density of GBs also renders these structures thermodynamically unstable under elevated temperatures. To overcome this, we employed density functional theory (DFT) calculations to identify boron as an optimal GB‐segregating element, based on its strongly negative segregation energy and favorable interfacial bonding characteristics. Moreover, DFT simulations further revealed that boron segregation has minimal impact on the energy barriers of the two rate‐determining steps in TCHOR, indicating that catalytic performance is preserved upon stabilization. To experimentally realize this design, boron was selectively introduced during NP attachment, allowing kinetically trapped, surface‐adsorbed boron species to localize at GBs. Advanced atomic‐scale characterization confirmed the spatially selective enrichment of boron at GBs and revealed a critical concentration threshold governing interfacial integrity. At concentrations exceeding the equilibrium segregation limit, boron accumulates excessively near GBs, introducing repulsive interactions that promote interfacial decohesion and structural degradation. In contrast, sub‐equilibrium levels of boron maintain GB cohesion during prolonged high‐temperature annealing, preserving both structural integrity and catalytic activity. These findings establish a generalizable three‐step framework for GB stabilization in nanocrystalline materials: 1) DFT‐guided selection of segregants with high GB affinity and minimal perturbation to catalytic energetics; 2) thermodynamic determination of segregation thresholds to avoid over‐segregation‐induced decohesion; and 3) kinetic trapping of targeted species during NP attachment to achieve spatially controlled interfacial modification. This strategy enables the fabrication of thermally stable, catalytically active, GB‐connected Pt nanograins, offering a versatile and scalable platform for the design of next‐generation nanocrystalline materials for catalysis and energy conversion.

## 2 Results and Discussion

### 2.1 Grain Boundary Engineering for Enhanced Catalytic Activity and CO Tolerance

**Figure 1:**
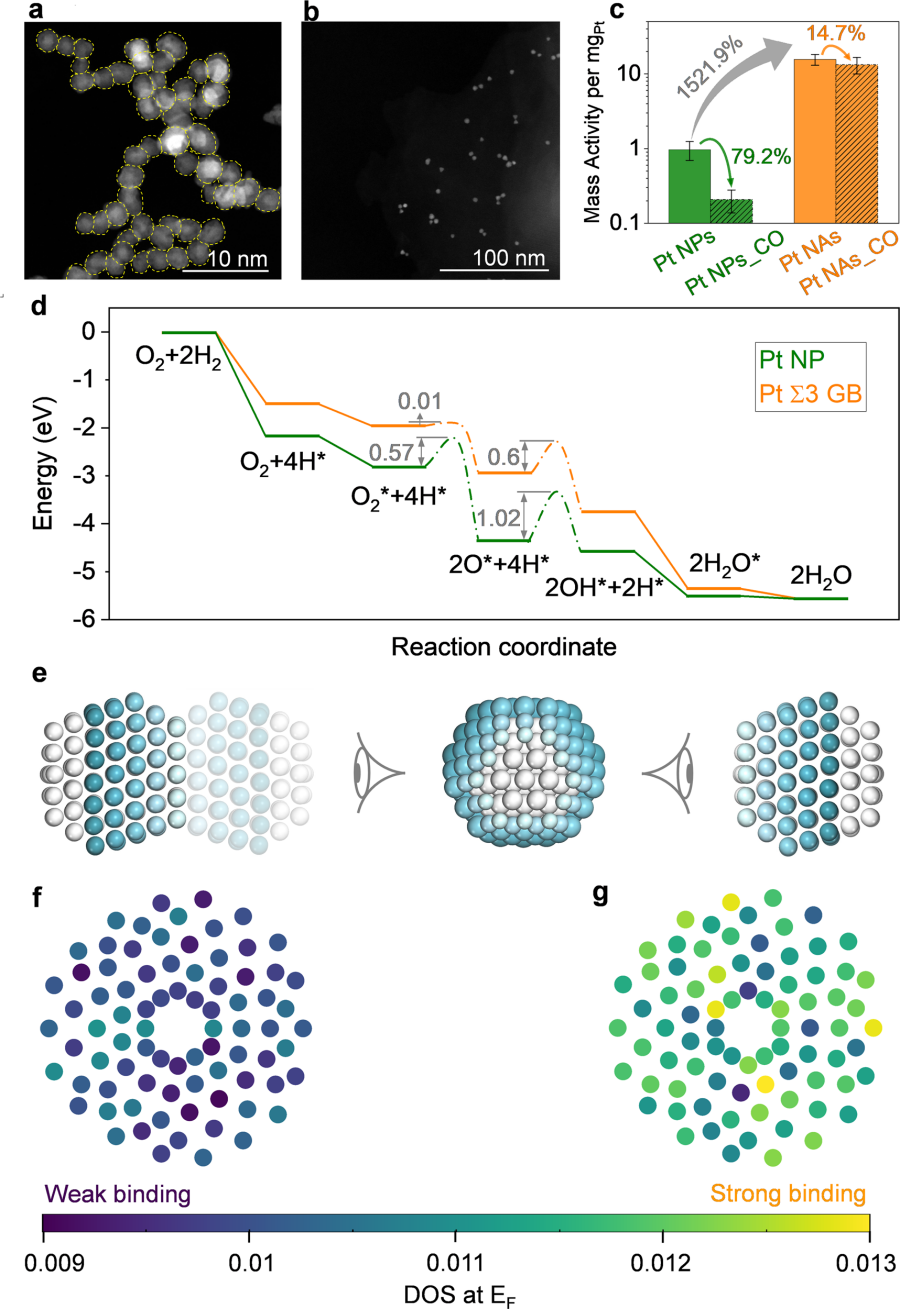
Comparison of isolated Pt NPs and GB‐connected Pt NAs in terms of structural features, catalytic activity, and theoretical simulations. a) HAADF‐STEM image of Pt NAs, with yellow dashed lines outlining the boundaries of individual NP building blocks. b) HAADF‐STEM image of citrate‐capped Pt NPs prior to the assembly process. c) Comparison of TCHOR catalytic activity of isolated citrate‐capped Pt NPs and GB‐connected Pt NAs during exposure to 4% H_2_ in air at ambient conditions. Equal amounts (1 mg) of Pt NPs and Pt NAs were deposited on J‐type thermocouples. As TCHOR is exothermic, catalytic activity was assessed by measuring the temperature rise upon H_2_ exposure (see Figure S6, Supporting Information). Prior to exposure, thermocouples were stabilized at room temperature (≈22 °C). Upon introduction of 4% H_2_, Pt NAs exhibited a sharp temperature increase to ≈400 °C, while Pt NPs showed only a slight rise. The greater temperature change in Pt NAs indicates significantly higher TCHOR activity, attributed to the lowered reaction barrier at GB sites. To quantitatively compare the TCHOR catalytic activity of Pt NPs and Pt NAs, the mass activity was calculated based on the temperature increase upon exposure to 4% H_2_. Specifically, mass activity per mg of Pt is defined as the temperature rise normalized to the baseline temperature and Pt loading: Mass activity (per mg Pt) = (T_4% H_2_ – T_initial)/(T_initial × 1 mg Pt). Take Pt NAs as an example, the temperature increases from ≈22 to ≈400 °C during 4% H_2_ exposure, yielding: Mass activity (per mg Pt) = (400–22 °C)/(22 °C × 1 mg Pt). This mass activity (per mg Pt) enables a direct and normalized comparison of TCHOR catalytic activity between Pt NPs and Pt NAs under identical conditions. To evaluate CO poisoning effects, measurements were also conducted with 1000 ppm CO (shaded region). Pt NAs maintained superior TCHOR activity and demonstrated enhanced CO tolerance compared to isolated Pt NPs. Error bars indicate the standard deviation from measurements taken at least three times. d) DFT‐calculated reaction energy profiles for TCHOR on a Pt NP model versus a Pt Σ3 GB model. The GB model displays substantially lower energy barriers for the key reaction steps in the TCHOR pathway: O_2_* + 4H* → 2O* + 4H* and 2O* + 4H* → 2OH* + 2H*, confirming GBs as the dominant active sites responsible for the superior TCHOR activity.^[^ ^44^ ^]^ e) Formation of a Σ3 GB through the connection of NPs with diameters of ≈2 nm. DFT simulations compare the electronic structure of five layers of surface atoms (80 atoms in total) near the GB with the corresponding surface atoms on an isolated NP. The side view of the Pt_383_ Σ3 [110] (111) GB model (left) and the Pt_201_ NP model (right) are displayed, with top views shown in the center along the indicated sightline. Surface atoms in the GB model are shaded in blue, with lighter shades representing atoms closer to the GB and darker shades indicating those farther away. Radially aligned integrated density of states at the Fermi level (DOS at *E*_F_) for each surface Pt atom of a Pt Σ3 GB (f) and an isolated Pt NP (g), visualized by projecting the five atomic layers onto concentric circles. Atoms closer to the GB are positioned nearer the center, while those farther from the GB are located toward the outer edge. The DOS at *E*_F_ for the GB model is 23–31% lower than that of the NP model, indicating fewer electronic states available for reactant interactions. This reduction in DOS at *E*_F_ correlates with weaker binding of reactants on the Pt surface, as evidenced by the CO binding energy: −1.9 eV for the isolated NP versus −0.9 eV for the Σ3 GB (Figure S11, Supporting Information). The reduced CO binding strength at the GB, approximately half that of the NP, suggests an enhanced anti‐CO poisoning effect. Further details on atomic positions are provided in Figure S10 (Supporting Information).

We engineered GBs (**Figure** **1**a; Figure S1, Supporting Information) by promoting the oriented attachment of ≈2 nm spherical Pt NPs (Figure 1b), resulting in the formation of a porous, 3D network of Pt nanoassemblies (Pt NAs, Figure S2, Supporting Information). The interconnected structure featured GBs with an average diameter of ≈1.5 nm (Figure S3, Supporting Information). Among the GBs evaluated (Figure S4, Supporting Information), ≈16% were classified as twin boundaries, while the remaining ≈84% were identified as high‐angle GBs, as determined by coincidence site lattice theory (Figure S5, Supporting Information).^[^ ^43^ ^]^ To elucidate the functional role of these GBs—particularly their impact on catalytic activity and thermal robustness—we employed thermal catalytic hydrogen oxidation (H_2_ + ½O_2_ → H_2_O, TCHOR) as a primary model reaction. TCHOR serves as a highly sensitive platform for probing both catalytic performance and structural evolution, owing to its strong dependence on GB structure.^[^ ^44^ ^]^ Moreover, the reaction is highly exothermic and capable of generating localized temperatures approaching ≈400 °C during exposure to 4% H_2_,^[^ ^44^ ^]^ allowing intrinsic evaluation of thermal durability under reaction‐relevant conditions. Additionally, since Pt‐based catalysts are highly susceptible to CO poisoning,^[^ ^45^, ^46^ ^]^ we introduced a controlled amount of CO into the TCHOR environment to assess anti‐poisoning behavior, mimicking realistic industrial hydrogen feedstocks.^[^ ^47^, ^48^, ^49^ ^]^

Catalytic testing revealed a pronounced difference in performance between isolated Pt NPs and their GB‐rich assembled counterparts (Figure 1c; Figure S6, Supporting Information). Both ligand‐capped and ligand‐free Pt NPs, lacking interparticle GBs, displayed low TCHOR activity (Figure 1c; Figure S7, Supporting Information), reflecting their intrinsically low surface reactivity. In stark contrast, Pt NAs—featuring abundant GBs between adjacent NPs—exhibited catalytic activity more than an order of magnitude higher (Figure 1c). Structural analyses show that isolated Pt NPs and GB‐rich Pt NAs share similar structural features (Figure S8, Supporting Information)—such as particle size and crystal facets—apart from the presence or absence of GBs. The pronounced enhancement in TCHOR activity observed in Pt NAs strongly suggests that GBs serve as high‐activity sites for the TCHOR. To mechanistically understand this observation, DFT calculations were conducted to examine the energy barriers associated with two key steps in the TCHOR pathway (Figure 1d): O_2_* + 4H* → 2O* + 4H* and 2O* + 4H* → 2OH* + 2H*. On the surface of isolated Pt NPs, the energy barriers for these steps were 0.57 and 1.02 eV, respectively. In contrast, when evaluated at Σ3 GBs, the barriers were significantly reduced to 0.08 and 0.60 eV. These results clearly demonstrate that GBs substantially lower the reaction energy landscape, thereby enhancing the overall catalytic efficiency of the Pt NAs. The TCHOR activity of single‐crystalline Pt nanowires—nearly free of GBs but with a geometry comparable to Pt NAs—was 38.5% higher than that of Pt NPs, indicating that a porous, nanowire‐shaped architecture provides only a modest enhancement (Figure S9, Supporting Information). By contrast, Pt NAs exhibited a 1521.9% increase in mass activity relative to Pt NPs (Figure 1c). This pronounced improvement shows that, while the porous nanowire geometry offers minor benefits, the dominant contribution arises from the high density of GBs, which furnish abundant catalytically active sites.

To assess the impact of GBs on CO tolerance, we carried out TCHOR measurements in the presence of 1000 ppm CO, a scenario relevant to industrial hydrogen streams.^[^ ^47^, ^48^, ^49^ ^]^ Isolated Pt NPs showed a dramatic 79.2% drop in catalytic activity, indicative of severe CO poisoning (Figure 1c). In contrast, Pt NAs maintained the majority of their performance, exhibiting only a 14.7% decline (Figure 1c). This contrast clearly demonstrates the superior CO tolerance conferred by nanoscale GB networks. The origin of this enhanced tolerance was explored through DFT calculations (Figure 1e–g; Figure S10, Supporting Information), which revealed substantial differences in electronic structure between GB‐connected and isolated Pt surfaces. Specifically, the density of states at the Fermi level for surface atoms at GB regions (Figure 1f) was found to be 20–30% lower than that of isolated Pt NPs (Figure 1g). This reduction implies fewer available electronic states for CO adsorption (Figure S11, Supporting Information). These theoretical predictions were experimentally validated using valence band photoemission spectroscopy, which showed a pronounced downward shift in the d‐band center—from −3.57 eV in isolated Pt NPs to −3.89 eV in GB‐rich Pt NAs (Figure S12, Supporting Information). This shift indicates a weakened interaction between the Pt surface and CO, offering a plausible explanation for the observed CO tolerance. Thus, the presence of nanoscale GBs mitigates one of the most persistent challenges in Pt catalysis: deactivation by CO.^[^ ^45^, ^46^ ^]^

### 2.2 Thermal Degradation of Grain Boundaries and Loss of Catalytic Function

**Figure 2:**
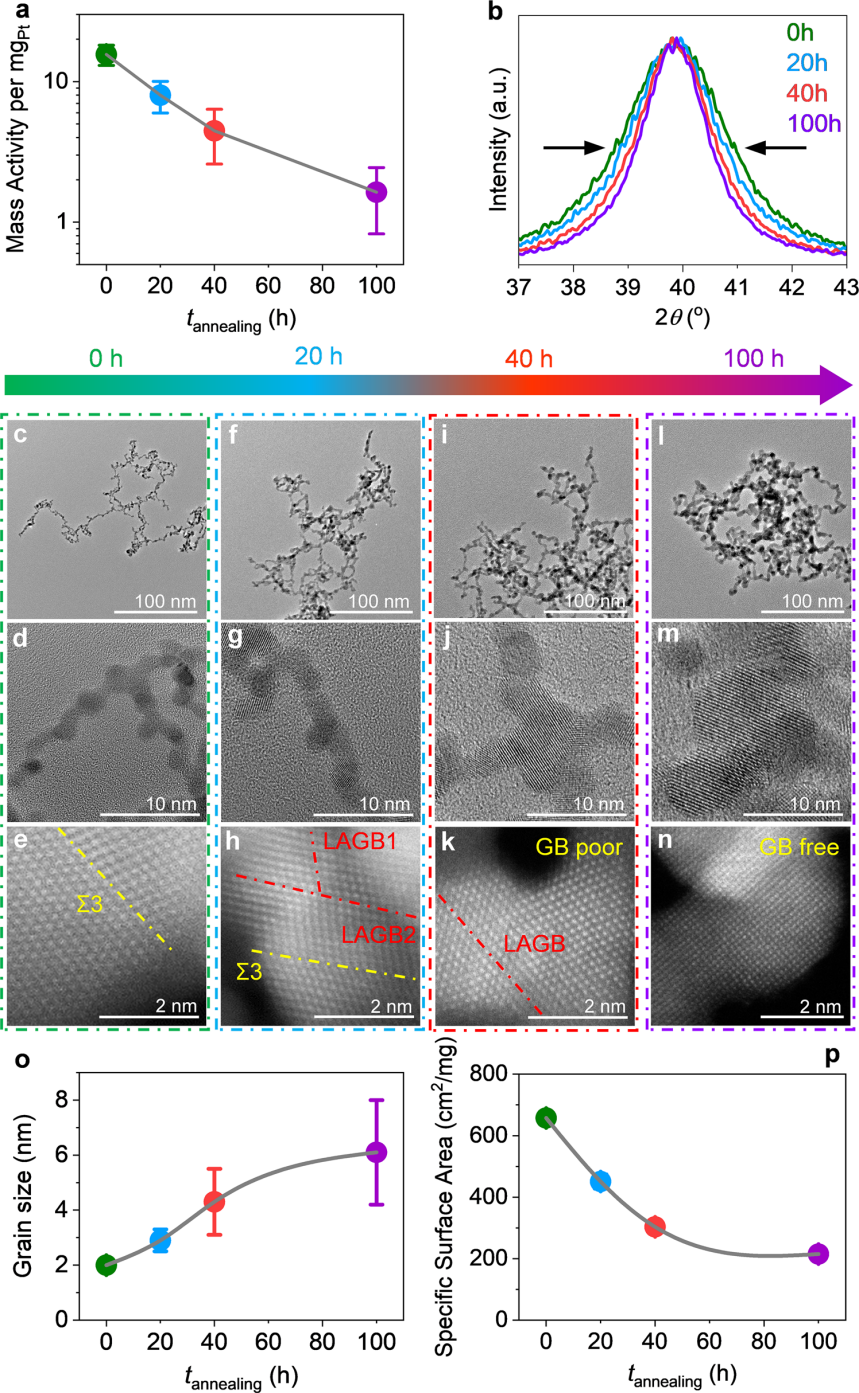
Structural evolution and activity degradation of B‐free Pt NAs during vacuum annealing at 400 °C. a) Evolution of TCHOR activity of B‐free Pt NAs as a function of vacuum annealing time at 400 °C. Error bars indicate the standard deviation from measurements taken at least three times. b) XRD patterns corresponding to the (111) facet of Pt NAs were recorded as a function of vacuum annealing time at 400 °C. c–n) TEM and HAADF‐STEM images of Pt NAs subjected to different annealing durations: 0 h (unannealed), 20, 40, and 100 h. For visual clarity, TEM images of unannealed, 20‐, 40‐, and 100 h‐annealed Pt NAs are highlighted with green, blue, red, and purple borders, respectively. c,d) Low‐ and high‐magnification TEM images of unannealed Pt NAs, showing the initial nanoparticle morphology and GB‐rich structure. e) HAADF‐STEM image of unannealed Pt NAs, illustrating abundant Σ3 GBs between adjacent nanoparticles. f,g) Low‐ and high‐magnification TEM images of Pt NAs after 20 h annealing, showing the onset of structural evolution. h) HAADF‐STEM image of 20 h‐annealed Pt NAs, revealing a noticeable reduction in Σ3 GBs and the formation of low‐angle GBs. i,j) TEM images of 40 h‐annealed Pt NAs, indicating further structural degradation. k) HAADF‐STEM image of 40 h‐annealed Pt NAs, showing the near disappearance of Σ3 GBs and predominance of low‐angle GBs. l,m) TEM images of 100 h‐annealed Pt NAs, displaying significant particle coarsening and GB loss. n) HAADF‐STEM image of 100 h‐annealed Pt NAs, confirming the complete disappearance of distinct GB features. o) Average grain size of Pt NAs as a function of vacuum annealing time at 400 °C, based on the analysis of over 100 grains per condition. p) The electrochemically active surface area of Pt NAs, determined from the hydrogen underpotential deposition region of cyclic voltammetry curves (Figure S14, Supporting Information), as a function of vacuum annealing time at 400 °C.

To probe the thermal stability of GB‐rich Pt NAs, we conducted vacuum annealing at 400 °C for up to 100 h, mimicking the high local temperatures encountered during TCHOR. Catalytic activity declined progressively—by 48.7%, 71.2%, and 89.7% after 20, 40, and 100 h, respectively—closely tracking structural evolution (**Figure** **2**a). X‐ray diffraction revealed a consistent narrowing of peak widths (Figure 2b), indicative of significant grain coarsening. Complementary high‐angle annular dark‐field scanning transmission electron microscopy (HAADF‐STEM) and high‐resolution TEM imaging confirmed a gradual increase in grain size (Figure 2c–o; Figure S13, Supporting Information), accompanied by a reduced exposed surface area (Figure 2p; Figure S14, Supporting Information). Atomic‐resolution HAADF‐STEM further showed that the as‐prepared Pt NAs featured a high density of GBs (Figure S4, Supporting Information), which diminished gradually during annealing (Figure 2h,k,n). High‐angle GBs transformed into low‐angle GBs and eventually into GB‐poor or GB‐free structures. As thermodynamically unstable, high‐energy interfaces, GBs are prone to migration, coalescence, and annihilation at elevated temperatures,^[^ ^19^ ^]^ depleting catalytically active sites. Since GB migration in nanoscale Pt is rapid—often completing within minutes once initiated^[^ ^19^ ^]^—the transient stage where one grain shrinks while another grows is hard to be captured in the ex situ measurements. As a result, the grain structures observed at each annealing time reflect the cumulative outcome of many such rapid events, manifesting as a monotonic increase in average grain size. This progressive loss of GBs directly accounts for the observed performance degradation, highlighting the intrinsic instability of GB‐rich architectures under thermal annealing. Our findings underscore the need for targeted strategies to preserve GB structures for long‐term operation in high‐temperature catalytic environments.

### 2.3 Effect of Boron Segregation on the Structural Stabilization and Catalytic Activity of Grain Boundaries

To address the thermal instability of GBs, we investigated hetero‐element segregation as a strategy for GB stabilization. While previous studies have demonstrated that certain elements can lower GB energy and enhance structural stability,^[^ ^50^, ^51^ ^]^ these efforts have been largely empirical, with limited insight into the underlying thermodynamics, spatial segregation behavior, or the interplay between stability and catalytic activity. A critical yet underexplored principle is that effective segregation should not only stabilize GBs but also preserve—or ideally enhance—the material's catalytic activity. However, a rational framework for engineering GB segregation to achieve both thermal robustness and functional efficiency remains undeveloped.

**Figure 3:**
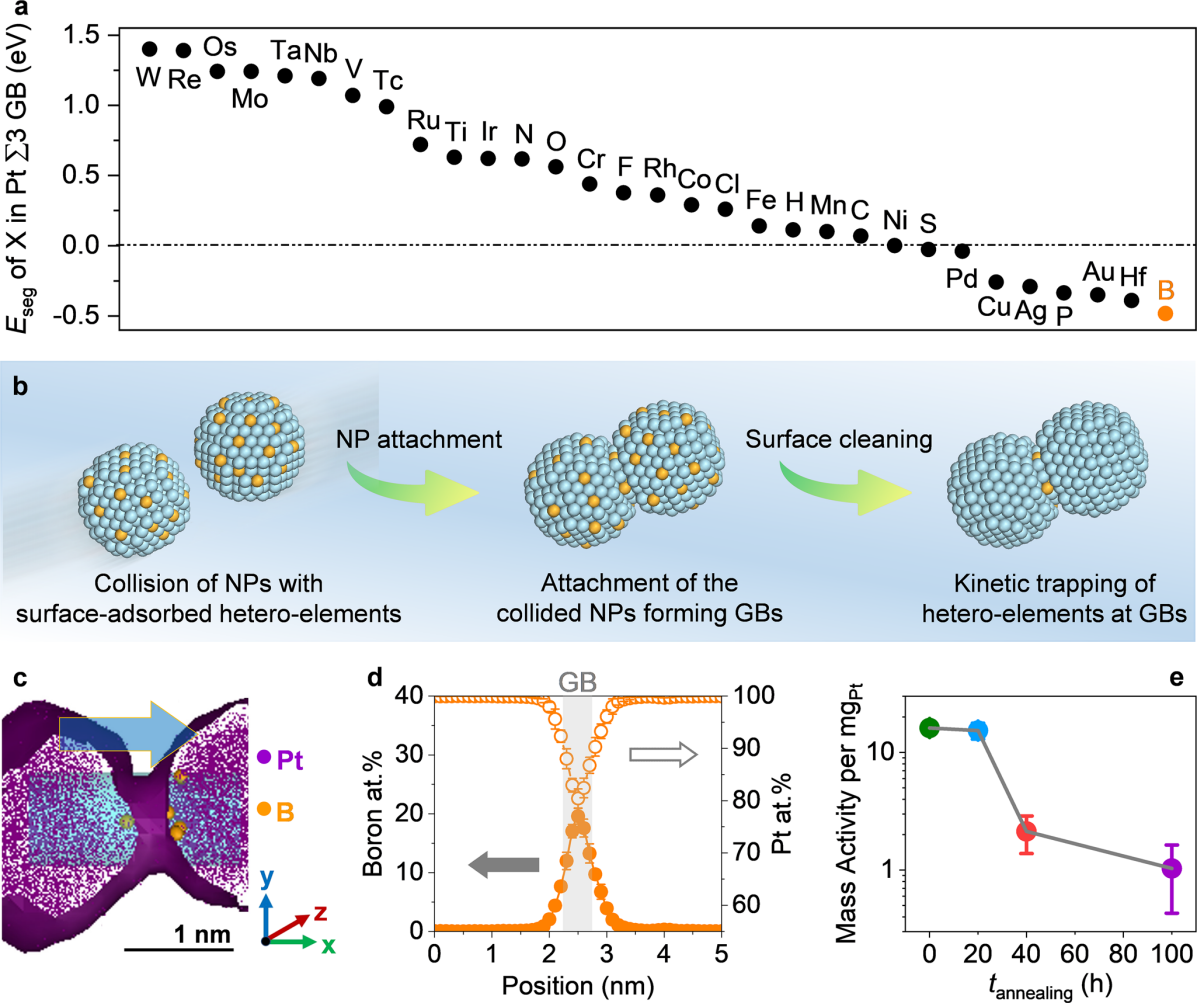
Design, implementation, and functional validation of controlled hetero‐element segregation at Pt GBs. a) DFT‐calculated segregation energies of various hetero‐elements at a Pt Σ3 GB, identifying those with strong thermodynamic driving forces for GB segregation. b) Schematic illustration of the controlled hetero‐element segregation strategy, consisting of three steps: 1) rapid collision of Pt NPs with hetero‐elements pre‐adsorbed on their surfaces, 2) oriented attachment of NPs to form GBs, during which pre‐adsorbed hetero‐elements are kinetically trapped at the interface, and 3) post‐attachment surface cleaning to remove unincorporated hetero‐elements, resulting in selective localization at the GBs. c) Representative GB tomogram of B‐Pt NAs, reconstructed from a 3D atom map acquired by atom probe tomography. Purple dots correspond to individual Pt atoms, while orange spheres denote individual B atoms; the larger sphere size for B atoms is chosen solely for visibility, given their much lower abundance compared with Pt atoms. The purple band depicts the Pt isodensity surface at a threshold of 150 atoms nm^−^^3^, delineating the outer contour of the NP building blocks. This threshold applies to all regions occupied by purple dots in the tomogram, not only those enclosed within the band. A cylindrical region of interest (ROI, *Φ*1.5 × 10 nm^3^) perpendicular to the GB is used to extract a 1D compositional profile of B, as illustrated in panel (d). d) Statistical analysis of the atomic concentration of B across more than 20 GBs based on 1D profiles from cylindrical ROIs. e) Evolution of TCHOR activity of B‐Pt NAs as a function of annealing time at 400 °C (0, 20, 40, and 100 h). Error bars represent standard deviations from at least three independent measurements.

To bridge this gap, we employed DFT simulations to screen 31 metallic and non‐metallic elements for their segregation energies (*E*_seg_) at Pt Σ3 GBs (**Figure** **3**a). A more negative *E*_seg_ signifies a stronger thermodynamic driving force for segregation.^[^ ^52^, ^53^, ^54^ ^]^ Among all candidates, six elements—Cu, Ag, P, Au, Hf, and boron (B)—exhibited strongly negative *E*_seg_ (Figure 3a), marking them as promising stabilizers. To determine whether segregation of these elements might compromise catalytic function, we calculated the energy barriers for the two rate‐limiting steps of the TCHOR at both pristine GBs and GBs segregated with each element. B‐segregated Pt GBs displayed energy barriers comparable to those of pristine GBs (Figure S15, Supporting Information), indicating that B segregation does not significantly affect reaction kinetics. In contrast, segregation of the other five elements led to substantial increases in the energy barriers (Figure S15, Supporting Information), which would be expected to slow the catalytic reaction. By integrating the criteria of strong segregation tendency and non‐negative impact on rate‐limiting step barriers, we identified B as the optimal segregation element for further investigation.

Building on these theoretical insights, we developed an experimental strategy to selectively localize B at Pt GBs. Pt NPs were first exposed to B‐containing species, followed by oriented attachment to form GB‐rich architectures (Figure 3b). This process kinetically trapped B species at GB regions formed between adjacent NPs. Subsequent washing removed surface B, ensuring localization within GBs. Atom probe tomography (APT), chosen for its atomic‐scale 3D resolution^[^ ^36^, ^39^, ^40^, ^55^, ^56^, ^57^, ^58^, ^59^, ^60^, ^61^, ^62^, ^63^ ^]^ and sensitivity to light elements,^[^ ^40^, ^64^, ^65^, ^66^, ^67^, ^68^ ^]^ revealed that B species were indeed concentrated within the GBs (Figure 3c; Figures S16–S18, Supporting Information). To quantify the extent of enrichment, 1D compositional profiles were measured perpendicular to the GB plane (Figure 3c,d). Since previous studies have shown that GB stability improves with increasing segregant concentration,^[^ ^42^ ^]^ we aimed to maximize B incorporation. By tuning the initial B coverage on NP surfaces prior to attachment, we achieved a maximum local B concentration of ≈20 at% within GBs (Figure 3d), yielding a sample referred to as B‐Pt NAs.

Catalytic testing revealed that B‐Pt NAs exhibited TCHOR activity comparable to B‐free Pt NAs prior to annealing, confirming that B segregation preserves the intrinsic catalytic function. However, upon annealing at 400 °C, B‐Pt NAs retained 95% of their activity after 20 h (Figure 3e)—substantially more than the 51.3% retention seen in B‐free Pt NAs (Figure 2a)—indicating that B segregation initially improves thermal stability. Unexpectedly, prolonged annealing for 40 h led to a sharp 87% decline in activity in B‐Pt NAs (Figure 3e), surpassing the 71.2% drop observed in B‐free samples (Figure 2a). This counterintuitive result suggests that B segregation does not universally stabilize GBs. While B may initially hinder GB migration, it may also induce structural instabilities over longer timescales. These findings reveal the complex, non‐monotonic role of segregants like B in governing GB dynamics and underscore the need for a more nuanced understanding of their long‐term effects in catalytic reactions.

### 2.4 Mechanistic Insights into the Stability–Instability Transition Induced by Boron Segregation

**Figure 4:**
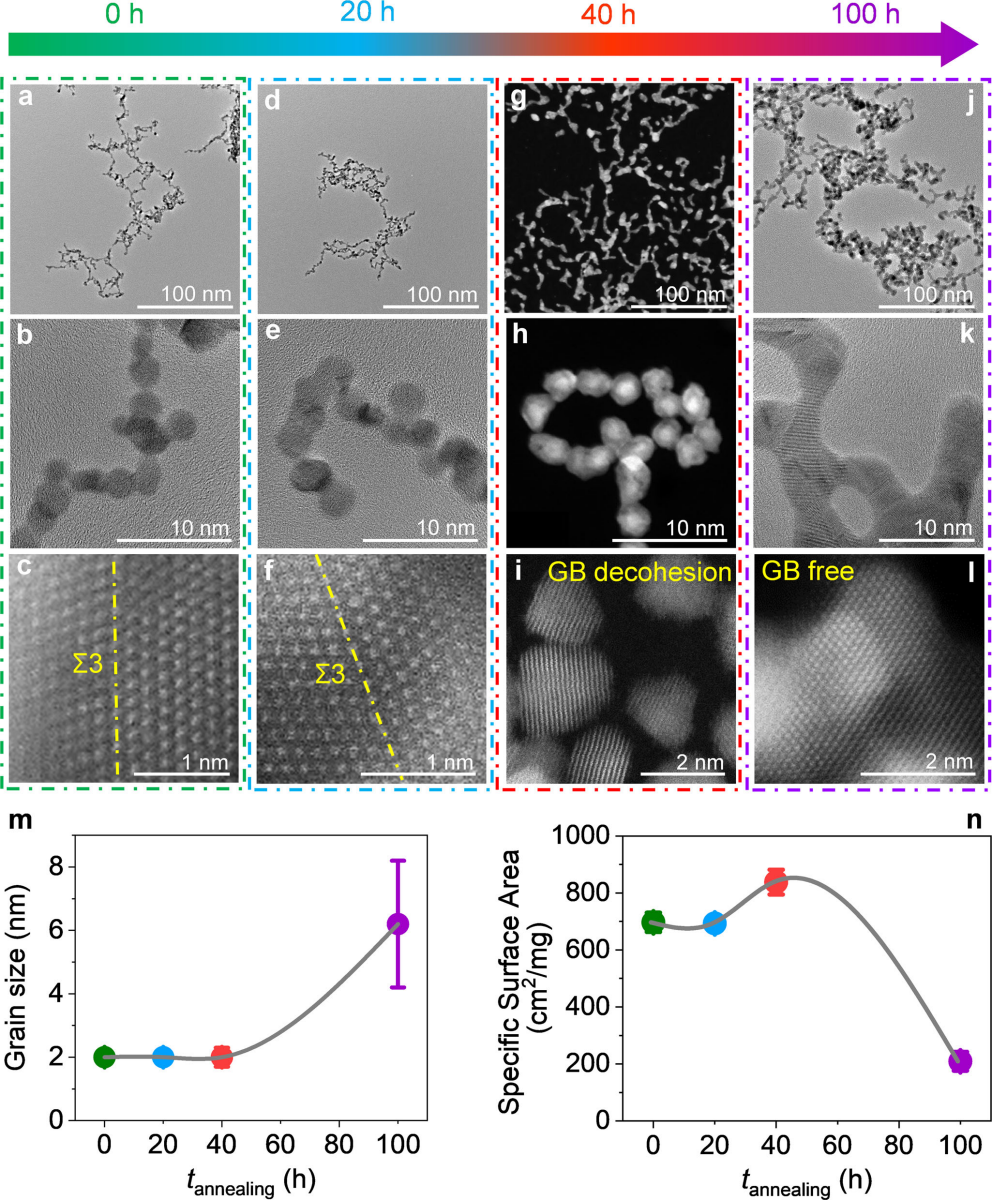
Structural evolution of B‐Pt NAs during vacuum annealing at 400 °C. a–l) TEM and HAADF‐STEM images of B‐Pt NAs after annealing for 0, 20, 40, and 100 h. For clarity, TEM images of 0‐, 20‐, 40‐, and 100 h‐annealed samples are marked with green, blue, red, and purple borders, respectively. a–c) High‐resolution TEM and HAADF‐STEM images of unannealed B‐Pt NAs, showing that nanoparticle building blocks are predominantly connected by Σ3 GBs. d–f) High‐resolution images of B‐Pt NAs after 20 h annealing, indicating that most Σ3 GBs are retained. g–i) Low‐ and high‐magnification HAADF‐STEM images after 40 h annealing, revealing widespread GB decohesion and the formation of gaps within the nanowire‐like network. j–l) High‐resolution TEM and HAADF‐STEM images after 100 h annealing, illustrating complete loss of GBs and transition to a GB‐free, coarsened structure. m) Evolution of average grain size as a function of annealing time, based on statistical analysis of more than 100 individual particles. n) Electrochemically active surface area of B‐Pt NAs, extracted from the hydrogen underpotential deposition region of cyclic voltammetry curves (Figure S20, Supporting Information), plotted against annealing time at 400 °C.

To understand this transition, we examined structural evolution during annealing. Up to 20 h, both the morphology and particle size of B‐Pt NAs remained largely unchanged (**Figure** **4**a–f,m), and the interconnected GB network was well preserved (Figure 4c,f). This initial period of apparent stability suggests that the GB structure is initially robust under thermal exposure. However, a marked transition was observed after 40 h of annealing (Figure 4g–i,m). At this stage, extensive GB decohesion and nanoparticle separation became evident, with clear gaps forming between previously interconnected NP building blocks (Figure 4g–i; Figure S19, Supporting Information). These features point to a collapse of the GB network, signaling a shift from gradual to more abrupt structural degradation. As annealing continued beyond 40 h, the separated NPs began to coalesce, forming significantly larger grains (Figure 4j–m). This coarsening process was accompanied by the near‐complete disappearance of GBs, and the particle size increased sharply.

These structural transitions were further supported by the evolution of the exposed surface area (Figure 4n; Figure S20, Supporting Information). After the initial 20 h of annealing, the surface area showed only a slight decrease, suggesting minimal structural change at this stage. However, after 40 h of annealing, the surface area unexpectedly increased, likely due to GB decohesion, which exposed additional surface regions. With further annealing up to 100 h, the exposed surface area dropped sharply, indicating the possible onset of particle agglomeration or significant grain coalescence. To capture these changes in real time, we employed in situ electrical resistance monitoring (Figures S21,S22, Supporting Information). This technique offers a sensitive and non‐invasive means of probing GB dynamic evolution, as electrical conduction within the network is primarily mediated through GB junctions. Any structural degradation—such as GB decohesion, migration, or elimination—disrupts electron transport and results in measurable changes in resistance. Consistent with the structural evolution, the resistance variation revealed three distinct regimes. In the first 20 h, resistance remained stable, reflecting the preservation of the GB network and uninterrupted electron transport (Figure S22, Supporting Information). Between 20 and 40 h, resistance increased sharply, eventually exceeding 120 MΩ (Figure S22, Supporting Information). This rise coincided with the onset of GB decohesion and the emergence of insulating gaps that disrupted conductive pathways across the network. Beyond 40 h, however, the resistance began to decline rapidly (Figure S22, Supporting Information). This drop indicates the gradual re‐establishment of conduction paths as the nanoparticles fused into larger grains with direct particle‐to‐particle contact. After 100 h of annealing, the resistance fell below that of the unannealed sample (Figure S22, Supporting Information), suggesting that many of the high‐resistance GB junctions had been eliminated, resulting in a more conductive—albeit structurally coarsened—network.

**Figure 5:**
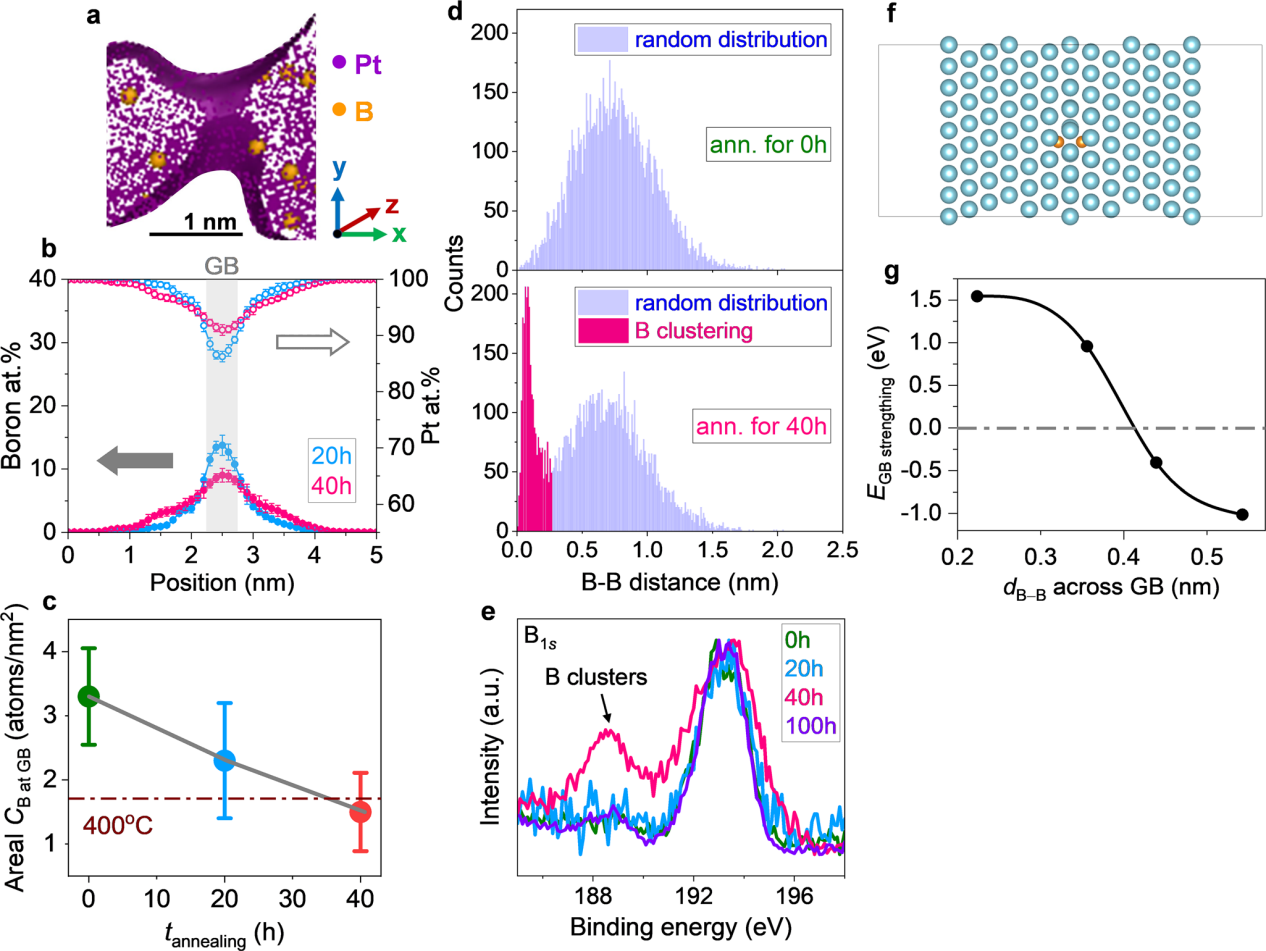
Evolution of boron spatial distribution in B‐Pt NAs during annealing. a) Representative GB tomogram of B‐Pt NAs after 40 h of annealing reconstructed from a 3D atom map obtained by atom probe tomography. Purple dots represent Pt atoms, and orange spheres denote B atoms, with the latter rendered larger for visibility due to their lower abundance. The purple band indicates the Pt isodensity surface at 150 atoms nm^−^^3^, outlining the NP building blocks. This threshold applies to all regions containing purple dots, not only those within the band. b) Statistical analysis of B atomic concentrations near GBs after 20 and 40 h of annealing at 400 °C, based on 1D profiles from over 20 GBs. c) Areal B concentration at GBs as a function of vacuum annealing time at 400 °C. The areal values are derived from the local B concentration at GBs by APT measurements. The dashed brown line indicates the theoretical equilibrium segregation limits at 400 °C, based on the McLean equation (see Supporting Information for details). d) Evolution of B distribution before and after 40 h of annealing at 400 °C. Prior to annealing, nearest‐neighbor B–B distance analysis shows a broad distribution centered above 0.5 nm (shaded blue), indicative of a random, dispersed state. After annealing, two distinct B–B populations emerge: a retained broad distribution above 0.5 nm (dispersed atoms) and a sharp new peak below 0.3 nm (shaded pink), signifying the onset of B clustering. e) High‐resolution XPS spectra of the B 1s region for B‐Pt NAs annealed at different times, with a new peak appearing at 40 h, indicating a change in the chemical environment of B. f) Top view of the slab model used for DFT calculations to evaluate the GB strengthening energy (*E*_GB strengthening_) of B‐segregated Pt Σ3 GBs. E_GB strengthening_ is defined as: *E*_GB strengthening _= (*E*_GB+B _− *E*_GB_) − (*E*_FS+B _− *E*_FS_), where E_GB_ and E_GB+B_ are the total energies of the clean and B‐segregated GBs, respectively, and E_FS_ and E_FS+B_ are the corresponding energies of the free surface. g) Calculated *E*_GB strengthening_ as a function of B–B distance across the GB. When B–B spacing exceeds 0.4 nm, *E*_GB strengthening_ is negative and becomes increasingly stabilizing with greater separation. Conversely, B–B distances below 0.4 nm lead to positive *E*_GB strengthening_, indicating repulsive interactions and destabilization. These results highlight the critical role of controlling interfacial B–B spacing to ensure effective GB stabilization.

The emergence of GB decohesion in B‐Pt NAs, absent in B‐free counterparts, points to the thermal redistribution of B as a key driver of structural instability during prolonged annealing. To explore this, we tracked B migration under thermal treatment (Figure S23, Supporting Information). At 400 °C, B atoms gradually diffuse from GBs into grain interiors (**Figure** **5**a), reducing their concentration at GBs over time (Figure 5b). This trend aligns with McLean's segregation theory,^[^ ^69^, ^70^, ^71^ ^]^ which predicts a temperature‐dependent decrease in the equilibrium GB concentration of segregants. To capture this redistribution more intuitively, we quantified GB enrichment using both local atomic percentage and areal concentration at GB (atoms nm^−^^2^), the latter offering a more direct measure of interfacial enrichment. Prior to annealing, B was highly enriched at GBs (≈3 atoms nm^−^^2^), far exceeding the equilibrium segregation limit of ≈1.7 atoms nm^−^^2^ for Pt Σ3 GBs at 400 °C (Figure 5c). This oversaturation creates a thermodynamic driving force for desegregation, prompting B to diffuse away from GBs. To resolve the atomic‐scale evolution of B near GBs, we performed nearest‐neighbor distance analysis using 3D atomic coordinates reconstructed by APT.^[^ ^72^, ^73^, ^74^, ^75^ ^]^ Before annealing, B–B distances near GBs were broadly distributed and centered above 0.5 nm (Figure 5d), consistent with random dispersion. After 40 h of annealing, the distribution split into two populations (Figure 5d): a broad peak remained above 0.5 nm, while a sharp new peak emerged below 0.3 nm, indicating the formation of B clusters. High‐resolution X‐ray photoelectron spectroscopy corroborated this clustering, revealing a new B 1s peak associated with B clusters (Figure 5e).^[^ ^76^, ^77^, ^78^ ^]^ These clusters likely originate from localized diffusion constraints imposed by intrinsic GB features—residual strain, dislocations, and interfacial discontinuities—that create confined regions for B accumulation (Figure S24, Supporting Information).^[^ ^41^, ^42^, ^44^ ^]^ Within these zones, B forms dense, nonequilibrium clusters with short interatomic distances, potentially initiating the GB decohesion observed in B‐Pt NAs.

To elucidate how B clustering influences GB stability, we performed DFT calculations to evaluate the interfacial energy of Pt Σ3 GBs as a function of B–B distance (Figure 5f). We introduced a descriptor—GB strengthening energy (E_GB strengthening_)—where negative values indicate stabilization and positive values reflect destabilization.^[^ ^79^ ^]^ The results revealed a threshold behavior: when B–B spacing exceeded 0.4 nm, E_GB strengthening_ was negative and increasingly favorable (Figure 5g), suggesting enhanced cohesion. However, when the spacing dropped below 0.4 nm, E_GB strengthening_ became positive and rose sharply with decreasing distance (Figure 5g), indicating that closely packed B atoms induce repulsive interactions and strain accumulation, ultimately destabilizing the GB. These predictions are consistent with our experimental observations. After 40 hours of annealing, B–B distances near GBs in B‐Pt NAs fell below 0.3 nm, well within the destabilizing regime identified by DFT. Such clustering is likely to disrupt Pt─Pt bonding across the interface, triggering GB decohesion.

### 2.5 Proof of Concept Showing that Rational Boron Segregation Enables Simultaneous Stabilization and Catalytic Preservation of Grain Boundaries

**Figure 6:**
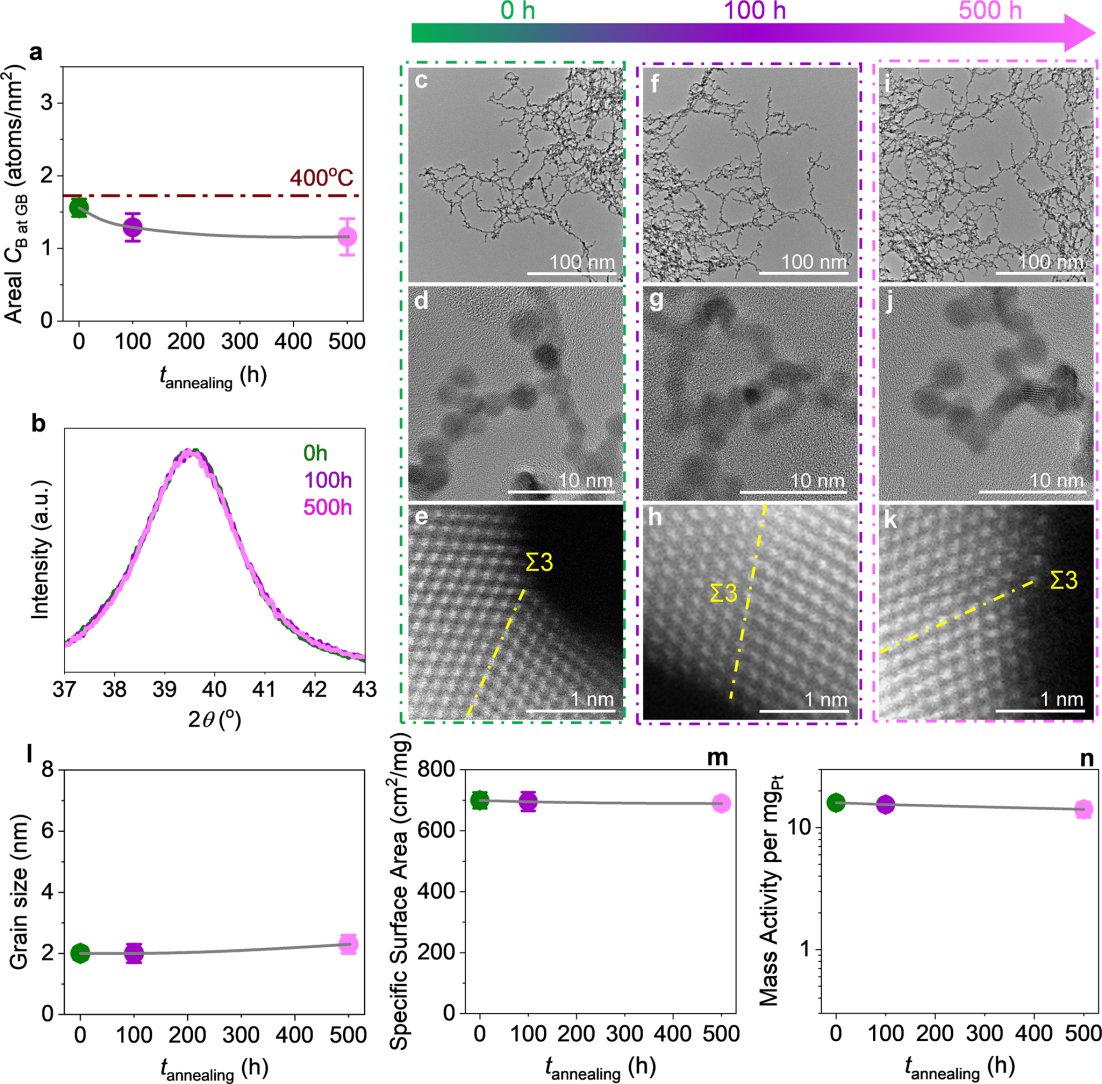
Proof of concept demonstrating rational boron segregation stabilizes BL‐Pt NAs during 500 h annealing at 400 °C, achieving long‐term thermal stability while maintain high catalytic activity. a) Areal boron concentration at GBs as a function of annealing time up to 500 h, measured by APT. Values are derived from local B concentrations across GBs (Figure **S25**, Supporting Information). The dashed brown line indicates the theoretical equilibrium segregation limit at 400 °C, estimated using the McLean equation. b) XRD patterns of the Pt (111) facet recorded as a function of annealing time. c–e) HR‐TEM and HAADF‐STEM images of unannealed BL‐Pt NAs. f–h) HR‐TEM and HAADF‐STEM images of BL‐Pt NAs after annealing of 100 h. i–k) HR‐TEM and HAADF‐STEM images of BL‐Pt NAs after annealing of 500 h. l) Average grain size of BL‐Pt NAs as a function of annealing time, based on analysis of >100 grains per condition. m) Electrochemically active surface area of BL‐Pt NAs, determined from the hydrogen underpotential deposition region of cyclic voltammetry (Figure **S26**, Supporting Information), as a function of annealing time. n) Evolution of TCHOR activity with annealing time (0, 100, and 500 h). Error bars represent standard deviations from at least three independent measurements.

**Figure 7:**
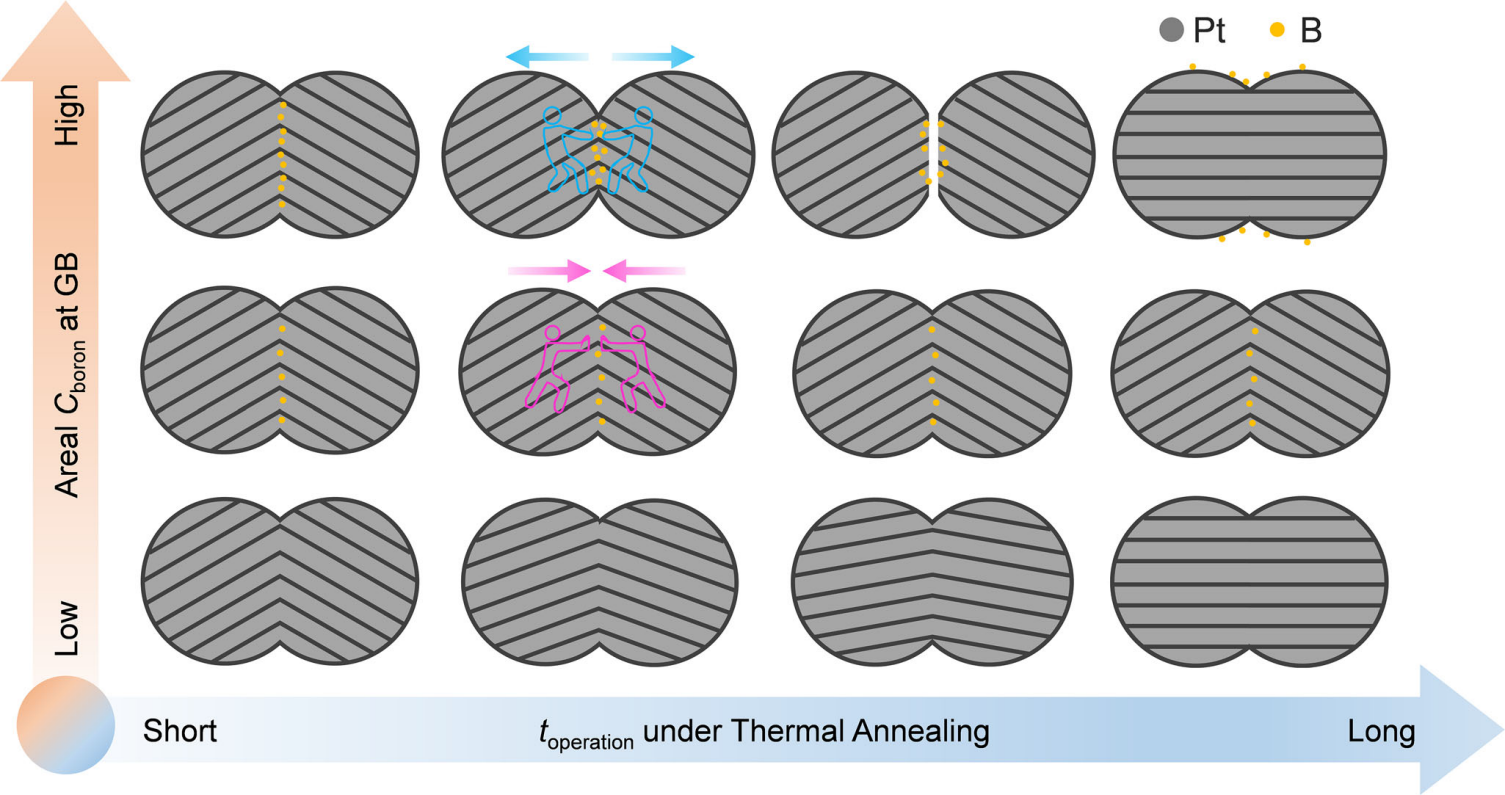
Schematic illustrating the influence of boron segregation on the stability of Pt GBs. The effect of boron depends critically on its areal concentration at the GB. When boron segregation exceeds the equilibrium limit, excess boron tends to diffuse away from the GB or accumulate on either side, weakening interfacial cohesion and potentially leading to GB decohesion. In contrast, when boron is present below the equilibrium segregation limit, it strengthens GB cohesion and enhances stability. Thus, the ability of boron to stabilize Pt GBs hinges on maintaining its concentration below the critical equilibrium threshold.

Based on these insights, we hypothesized that long‐term GB stability requires B enrichment to remain below the equilibrium segregation limit (**Figure** **6**a). To test this, we synthesized a B‐segregated Pt NAs sample with an areal B concentration at GBs of ≈1.5 atoms nm^−^^2^ (Figure 6a; Figure S25, Supporting Information)—slightly below the predicted equilibrium segregation limit—denoted as BL‐Pt NAs. Remarkably, after 500 h of annealing at 400 °C, BL‐Pt NAs showed no significant changes in XRD peak widths (Figure 6b), morphology (Figure 6c–k), average grain size (Figure 6l), GB structure (Figure 6e,h,k), or exposed surface area (Figure 6m; Figure S26, Supporting Information). Catalytic testing revealed only a 6.7% loss in activity (Figure 6n), demonstrating exceptional thermal stability. These contrasting outcomes illustrate the dual role of segregation (**Figure** **7**): while sub‐equilibrium B levels reinforce GB cohesion (BL‐Pt NAs), excess B leads to desegregation and cluster formation (B‐Pt NAs), which undermine structural and functional stability. Thus, segregation of the same species at GBs can either stabilize or destabilize the interface, depending critically on whether its local concentration exceeds the equilibrium segregation limit (Figure 7).

Building on these findings, we propose a three‐step framework for rational GB stabilization that preserves high catalytic activity. First, DFT is used to screen for elements with strong segregation tendencies that do not significantly alter the energy barriers of rate‐determining catalytic steps. Second, the McLean equation is applied to estimate the maximum equilibrium segregation limit, beyond which structural instability may arise. Third, a surface‐mediated kinetic trapping strategy enables precise control over segregant incorporation during NP attachment, selectively enriching GBs while avoiding oversaturation. We validated this approach in BL‐Pt NAs, where all three criteria were met: boron was identified via DFT as a strong GB segregant with minimal impact on catalytic energetics, its concentration was maintained below the equilibrium threshold, and oriented NP attachment directed B species—initially adsorbed on NP surfaces—specifically into GB regions. The resulting material retained both structural integrity and catalytic activity after 500 h at 400 °C. In contrast, exceeding the segregation limit led to GB collapse and a sharp decline in performance, underscoring the critical role of controlled segregation in achieving long‐term stability without compromising function.

Together, our findings uncover the mechanistic origins of GB (de)stabilization through segregation and establish a broadly applicable design strategy for engineering durable, high‐performance polycrystalline nanomaterials. This approach opens a path toward rational interface engineering in a broad class of functional materials where reactivity and durability must be optimized in tandem.

## 3 Conclusion

Here, we demonstrated that grain boundaries (GBs) serve as highly active and CO‐tolerant catalytic sites in platinum nanoassemblies (Pt NAs). Assembling ≈2 nm Pt nanoparticles into interconnected networks enriched with Σ3 GBs led to marked improvements in thermal catalytic hydrogen oxidation compared to isolated nanoparticles. DFT calculations and spectroscopic analyses revealed that GBs modulate the local electronic structure, lowering reaction barriers and suppressing CO adsorption—key factors behind their enhanced activity and resistance to poisoning. These advantages also translated to the methanol oxidation reaction (MOR) in acidic media (Figure S27, Table S1, Supporting Information), where GB‐rich Pt NAs exhibited a 293.4% increase in activity and sustained performance with only an 8.3% decline under 1000 ppm CO, compared to a 73.8% drop for isolated Pt NPs.

Yet, these benefits come with a critical challenge: GBs are thermally unstable, prone to coarsening and degradation that undermine long‐term performance. Solute segregation is often considered a straightforward remedy, but our findings challenge this assumption. Using boron as a model segregant, we uncover a striking duality—segregation can either stabilize or destabilize GBs depending solely on local concentration. When boron levels exceed the equilibrium segregation limit, it migrates and clusters near the interface, generating repulsive forces that drive decohesion and nanoparticle separation. In contrast, carefully controlled sub‐equilibrium segregation preserves GB integrity even after extended high‐temperature annealing.

These results overturn the long‐standing notion that segregation is commonly beneficial. We demonstrate that GB stabilization requires more than the right choice of element—it demands precise atomic‐level control over segregant chemistry, concentration, and spatial distribution. To address this challenge, we propose a three‐step segregation engineering strategy: 1) computational screening identified elements with strong GB stabilization (negative segregation energy) and minimal impact on catalytic activity (low reaction barriers), 2) thermodynamic estimation via the McLean equation to define appropriate segregant concentration windows, and 3) selective incorporation of segregants through kinetic trapping during nanoparticle attachment, exploiting surface adsorption prior to attachment. This integrated strategy bridges theory and experiment, enabling deliberate, controllable GB stabilization that preserves catalytic functionality under realistic operating conditions. Beyond platinum, the principles we establish are generalizable across material systems and interfaces.

Altogether, this work challenges conventional assumptions and opens a new paradigm in materials design—one where GBs are not merely tolerated but intentionally activated and stabilized. By leveraging the dual role of GBs as both catalytic centers and tunable structural motifs, we pave the way for robust, high‐performance nanomaterials that meet the growing demands of next‐generation catalysis, energy conversion, and functional device applications.

## 4 Experimental Section

### Material Synthesis

Oleylamine (OAm)‐capped Pt nanoparticles (NPs) were synthesized as follows: A 9 mL solution of OAm (27.4 mmol) was degassed under argon at 80 °C for 20 min in a 25 mL round‐bottom flask. Subsequently, 0.051 mmol (20 mg) of platinum(II) acetylacetonate (Pt(acac)_2_) was added, and the mixture was vigorously stirred magnetically for 30 min to form a homogeneous Pt(acac)_2_‐OAm complex. The flask was then heated in an oil bath preheated to 210 °C, achieving this temperature within 10 min. Carbon monoxide (100 sccm) was bubbled through the mixture for 1 min. After removing the flask from the oil bath, it was allowed to cool naturally. The resulting OAm‐capped Pt NPs were precipitated with ethanol by centrifugation and redispersed in cyclohexane, with this process repeated thrice. Finally, the OAm‐capped Pt NPs were dispersed in 5 mL of cyclohexane for subsequent ligand exchange to citrate.

To prepare citrate‐capped Pt NPs, a two‐step ligand exchange was performed. First, 5 mL of OAm‐capped Pt NPs were mixed with 5 mL of diethanolamine (DEA) and stirred for 24 h. The solution was centrifuged, washed with ethanol, and redispersed in 10 mL of DEA, followed by another 24 h of stirring to fully replace OAm with DEA. The DEA‐capped Pt NPs were then washed with ethanol and redispersed in 5 mL of deionized (DI) water. In the second step, to the 5 mL aqueous suspension of DEA‐capped Pt NPs, 1 mL of 10 mg mL^−1^ sodium citrate and 1 mL of 10 mg mL^−1^ boric acid were added, followed by 1 h of sonication and 24 h of magnetic stirring. For B‐segregated Pt NAs with B–B distances of 0.7 or 1.5 nm, 1 mL of 20 or 10 mg mL^−1^ boric acid, respectively, was used. For B‐free Pt NAs (BF‐Pt NAs), 1 mL of 10 mg mL^−1^ citric acid was used. The resulting citrate‐capped Pt NPs were washed and redispersed in DI water.

The citrate‐capped Pt NP solution underwent dialysis to remove impurity ions. Placed in dialysis tubing suspended in a 1 L beaker of DI water with magnetic stirring, the DI water was refreshed twice daily over three days. A flow of high‐purity hydrogen gas (200 sccm) was bubbled through a 10 mL solution of ≈3.6 µm citrate‐capped Pt NPs for 5 h. The color change from grayish‐brown to clear indicated the formation of B‐segregated Pt NAs. After stopping the H_2_ gas flow, the solution settled for 2 h to allow the B‐segregated Pt NAs to sediment. The supernatant was carefully removed, and the B‐segregated Pt NAs were thoroughly washed with DI water. BF‐Pt NAs were prepared similarly, excluding the addition of boric acid.

### Material Characterization: Transmission Electron Microscopy Characterization

HAADF‐STEM images were acquired using a Thermo Fisher Titan Themis 60–300 microscope operating at 300 kV. High‐resolution transmission electron microscopy images were obtained with aberration correction. Particle size distribution was analyzed using Nano Measurer 1.2 software. Gatan Microscopy Suite 3.0 (GMS 3.0) analyzed HAADF‐STEM images, identifying crystallographic planes and GBs by evaluating interplanar spacings and angles from Fast‐Fourier transformed images.

### Atom Probe Tomography (APT) Characterization

Pt NAs were co‐electrodeposited into a Ni matrix. A Cu foil (0.2 cm^2^) was used as the working electrode, etched with 0.5 m H_2_SO_4_, and a Pt mesh (2 cm^2^) as the counter electrode. The electrolyte contained a 5 mL aqueous nickel solution with 1.5 g NiSO_4_·6H_2_O, 0.225 g citric acid, and 10 mg of Pt NAs, sonicated for 1 h. A constant current of −19 mA was applied to the working electrode for 500 s to co‐encapsulate Pt NAs within the Ni film on the Cu foil. Needle‐shaped APT specimens were prepared using a Ga‐plasma focused ion beam (FEI 600 DualBeam). The Ni film with embedded Pt NAs exhibited surface protrusions, which were sectioned and transferred onto Si coupons as lamellas. These lamellas were cross‐sectionally analyzed and sharpened into needle‐shaped APT specimens, then analyzed using a LEAP 5076 XS instrument (Cameca) in pulsed laser mode. To ensure the accuracy of the 3D reconstruction and precise spatial localization of B atoms, APT specimens were prepared using a FEI Helios 600 DualBeam FIB‐SEM system, where sharp needle‐shaped tips were fabricated from the Pt NAs. Key geometrical parameters—specifically the tip apex radius and shank angle—were measured for each tip using high‐resolution SEM imaging and directly input into the IVAS 3.8.4 reconstruction software to match the physical geometry of the specimen. The 3D reconstruction was performed based on time‐of‐flight mass spectrometry for elemental identification and a field evaporation model for determining spatial coordinates. Depth (*z*‐axis) resolution was dynamically calibrated using ion detection rates and evaporation history, while lateral (*x–y*) coordinates were refined using detector hit positions and lattice‐based corrections derived from the known crystallographic structure of Pt. This combination of experimentally calibrated inputs and lattice‐guided refinement minimized spatial distortion and ensured high‐resolution reconstruction of atomic positions. As previously demonstrated in the studies of GB segregation in Pd‐based nanogels, APT is highly effective in detecting and localizing light elements, such as boron, at the atomic scale.

### Other Characterizations

High‐resolution X‐ray diffraction patterns were recorded using a Bruker powder X‐ray diffractometer with Cu Kα radiation. X‐ray photoelectron spectroscopy spectra were acquired with an Al Kα X‐ray Photoelectron Spectrometer (Thermo Scientific), calibrated using the C1s peak at 284.8 eV. Pt concentration in the ink and the dissolved Pt concentration of Pt NAs/C after 50 000 cycles of MOR accelerated durability tests (ADTs) were measured by inductively coupled plasma mass spectrometry.

### Monitoring Electrical Resistance Variation during Annealing at 400 °C in Vacuum

Due to the propensity of electrons to scatter at GBs, GB‐rich materials exhibited higher electrical resistance compared to GB‐free materials. As GB density decreased, the resistance correspondingly diminished. Therefore, monitoring the resistance of GB‐rich materials could provide insights into structural changes. Wet slurries of Pt NAs were prepared by mixing 1 mg of Pt NAs with 1 mL of ethanol. These slurries were spin‐coated onto sensor substrates to fabricate Pt NAs‐coated sensors. To assess the thermal stability of Pt NAs, the sensors were annealed at 400 °C in a vacuum. Electrical resistance variations of the Pt NAs‐coated sensors during annealing were recorded using a data acquisition meter (Keysight/Agilent 34972A LXI). The sensor substrates, composed of sintered alumina with interdigitated gold electrodes on one side, served as electrical insulators. The distance between adjacent Au electrodes was 0.1 mm. Prior to spin coating, the substrates were thoroughly cleaned with deionized water and ethanol, followed by drying. In some regions, gaps between the interdigitated Au electrodes were bridged by single Pt NAs. In these cases, any structural changes in the Pt NAs would be directly reflected in variations in electrical resistance.

### Thermal Catalytic Hydrogen Oxidation Reaction Measurement

At ambient conditions, O_2_ gas dissociated into O atoms at GBs with diameters less than 4 nm, which subsequently reacted with H_2_ gas to form H_2_O. This reaction did not occur in GB‐free regions under the same conditions. The exothermic nature of this reaction allowed for the assessment of thermal catalytic hydrogen oxidation activity through temperature variation measurements. To measure these temperature changes, wet slurries of Pt NAs (1 mg Pt NAs in 100 µL ethanol) were drop‐casted onto a J‐type thermocouple wire (Keysight Agilent, Part number: 34 970–61 606, measuring range: −40 to 750 °C) and air‐dried. The tests were conducted using a custom‐built apparatus at ambient conditions, comprising synthetic air and H_2_ gas cylinders, mass flow controllers (Bronkhorst), a data acquisition meter (Keysight/Agilent 34972A LXI) for real‐time temperature recording, and a PC for data storage. The Pt NAs‐coated J‐type thermocouple wire was positioned directly in front of the gas outlet during the test. Before H_2_ exposure, the Pt NAs‐coated thermocouple wire was stabilized in synthetic air (21% O_2_ + 79% N_2_) at a flow rate of 1000 sccm. H_2_ gas was then mixed with synthetic air to achieve varying H_2_ concentrations (4%, 3%, 2%, 1%, 0.5%, 0.25%, 0.1%, and 0.05%). These H_2_/air mixtures sequentially flowed over the thermocouple wire at a constant rate of 1000 sccm, allowing the temperature variation curves of the Pt NAs‐coated thermocouple wire to be obtained. Upon H_2_ exposure, the temperature increased rapidly from room temperature (≈22 °C) to higher values, such as ≈370 °C for a 4% H_2_ concentration in air. When H_2_ was removed, the temperature quickly returned to room temperature. The reaction activity was calculated as the ratio of the temperature change (Δ*T*) to the initial temperature (22 °C) of the Pt NAs‐coated thermocouple wire upon H_2_ exposure.

To ensure the reproducibility and reliability of the catalytic performance data, all tests were conducted on at least three independently synthesized batches for each catalyst type (Pt NPs, Pt NAs, B‐Pt NAs, and BL‐Pt NAs). These batches were prepared at different times using separate precursor lots to ensure batch‐to‐batch independence. Catalytic measurements were repeated under identical conditions for each batch, and the reported values represent averaged results with corresponding standard deviations.

### Electrochemical Measurements

Electrochemical measurements were conducted using a CHI660E electrochemical workstation at ambient conditions. Glassy carbon electrodes (diameter = 5 mm, geometric area = 0.196 cm^2^), Pt wire, and Ag/AgCl (3 m KCl) were used as the working, counter, and reference electrodes, respectively, with potentials converted to the reversible hydrogen electrode (RHE). Calibration of the Ag/AgCl electrode was performed in H_2_‐saturated 0.1 m HClO_4_ with polished Pt wires as working and counter electrodes, calculating RHE potential as E(RHE) = E(Ag/AgCl) + 0.269 V. Pt NAs were sonicated in DI water for 1 h, followed by adding commercial Vulcan carbon (XC‐72) with a mass four times that of Pt (wt.% Pt = 20%) and further sonication for 1 h to form Pt samples/C. The mixture was dispersed in isopropanol and Nafion (5%) (v:v = 1:0.005) and sonicated for 1 h to create a homogeneous ink with Pt concentration controlled at 0.25 mgPt mL^−1^ by ICP‐AES. A uniform catalyst layer was prepared by pipetting 5 µL of the ink onto the glassy carbon electrode and drying at ambient conditions, achieving a Pt loading of 6.4 µg cm^−^^2^.

Cyclic voltammetry (CV) tests were conducted in N_2_‐saturated 0.1 m HClO_4_ at a scan rate of 50 mV s^−1^. The electrochemical active surface area (ECSA) was calculated by integrating the hydrogen adsorption charge on CV curves, assuming 210 µC cm^−^^2^ for a hydrogen monolayer. MOR tests were performed in N_2_‐saturated 0.1 m HClO_4_ and 0.1 m methanol, sweeping from 0.05 to 1.25 V versus RHE at 50 mV s^−1^. Current densities were normalized to the geometric area of the glassy carbon electrode (0.196 cm^2^), with specific and mass activities calculated by normalizing the current to the ECSA and Pt mass, respectively. ADTs were performed by cyclic potential sweeps from 0.05 to 1.25 V versus RHE at 100 mV s^−1^ for 50 000 cycles in N_2_‐saturated 0.1 m HClO_4_ and 0.1 m methanol at room temperature. Post‐50 000 cycle Pt concentrations in the electrolyte were measured by ICP‐AES to assess Pt NAs dissolution.

### DFT Calculations

DFT calculations were conducted using the Vienna ab initio simulation package employing the projected‐augmented wave method with a 400 eV basis set cutoff.^[^ ^80^, ^81^, ^82^ ^]^ The generalized gradient approximation was utilized to assess exchange‐correlation energy, employing both the Perdew–Burke–Ernzerhof (PBE) and the revised PBE functional by Hammer et al. (RPBE).^[^ ^83^, ^84^ ^]^ Brillouin zone sampling employed the *Γ*–centered Monkhorst–Pack scheme.^[^ ^85^ ^]^ The computational details are provided in the Supporting Information.

## Conflict of Interest

The authors declare no conflict of interest.

## Author Contributions

X.G. and B.G. conceptualized the study and designed the research framework. X.G. led the study, performing sample preparation, microstructural analyses, and mechanism understanding. X.L. helped with the microstructural analyses, while Z.W. provided support with theoretical simulations. The observations were collectively discussed and interpreted by X.G., X.L., Z.W., and B.G. The manuscript was drafted by X.G., Z.W., and B.G., with all authors reviewing and approving the final version prior to submission.

## Supporting information



Supporting Information

## Data Availability

The data that support the findings of this study are available from the corresponding author upon reasonable request.
