# Supplementary material for: Rational Grain Boundary Segregation Enables Long‐Term Thermally Stable, Catalytically Active, and CO‐Tolerant Nanograined Metals
Source: Adv Sci (Weinh). 2025 Sep 20;12(44):e12730. doi: 10.1002/advs.202512730 (PMC12667514; doi:10.1002/advs.202512730)
Supplement: Supplementary file 1 — Supporting Information [file ADVS-12-e12730-s001.pdf]

Rational Grain Boundary Segregation Enables Long-Term Thermally Stable, Catalytically Active, and CO-Tolerant Nanograined Metals

Xin Geng,[1*] Xiaolong Lu,[2] Zhenyu Wang,[1*] Baptiste Gault,[1,3*]

1 Max Planck Institute for Sustainable Materials, Max-Planck-Straße 1, 40237 Düsseldorf, Germany

2 School of Intelligent Manufacturing Ecosystem, Xi’an Jiaotong-Liverpool University, 215123 Suzhou, China

3 Department of Materials, Royal School of Mines, Imperial College London, SW7 2AZ, UK

* Corresponding author. Email: x.geng@mpie.de, z.wang@mpie.de, b.gault@mpie.de

Computational Details

Density functional theory (DFT) calculations were performed using the Vienna Ab initio Simulation Package,[1,2] employing the projected-augmented wave method for core electron description.[3] A basis set cutoff of 400 eV was used for standard calculations,[4] while a higher cutoff of 500 eV was applied for electronic structure calculations to ensure accuracy. The revised Perdew-Burke-Ernzerhof exchange-correlation functional, based on the generalized gradient approximation,[5,6] was utilized throughout.

The Pt (111) surface was modeled using a four-layer slab, with the bottom two layers fixed and the upper layers and adsorbed molecules relaxed. A vacuum layer of 14 Å was introduced to avoid interactions between periodic images. The Brillouin zone was sampled using a 2×2×1 Γ-centered k-point grid for Pt (111) slab optimizations, while only the Γ point was considered for isolated Pt_{201} NP and Pt_{383} Σ3 [110] (111) GB models. The convergence criteria for energy and force during structural optimization were set to 10^{-6} eV and 0.01 eVÅ^{-1}, respectively.

For high-resolution density of states (DOS) calculations, a Gaussian smearing of 0.02 eV was employed, with 3000 grid points used to sample the energy range from -20 eV to 20 eV. The analysis focused on the electronic states near the Fermi level (E_F) for surface Pt atoms in both the Pt_{201} NP and the Pt_{383} Σ3 GB models. States within an energy window of ±1 eV around E_F were selected for the evaluation, as these states are most relevant for surface chemical interactions. Given that electronic states above E_F are unoccupied, the integration was performed over the range from E_F–1 eV to E_F, according to the following expression:

$$\text{Integrated DOS} = \int_{E_{Fermi-1}}^{E_{Fermi}} \text{DOS}(\text{Pt}_i)$$

where DOS (Pt$_i$) represents the electronic density of states for the i-th Pt atom on the surface. This approach provides insight into the distribution of available electronic states near the Fermi level, which are crucial for understanding the reactivity of the Pt surfaces.

The binding energy of a CO molecule ($E_{CO\ binding}$) on different Pt models was calculated using the following expression:

$$E_{CO\ binding} = E^{Y}_{Pt+CO} - E^{Y}_{Pt} - E_{CO}$$

where Y denotes the specific model used in the calculation: the Pt (111) surface slab, the Pt_{201} NP, or the Pt_{383} Σ3 GB. In this equation, E^{Y}_{Pt+CO} represents the total energy of the Pt model with the adsorbed CO molecule, while E^{Y}_{Pt} is the total energy of the corresponding Pt model without the CO molecule. E_{CO} is the total energy of an isolated CO molecule in the gas phase. This approach allows for a quantitative assessment of CO adsorption strength on different Pt surfaces, providing insights into the variations in anti-CO poisoning behavior across distinct Pt structural motifs.

To assess the segregation behavior of hetero-elements at the Pt Σ3 GB, the segregation energy (E_{seg}) was calculated using DFT. Taking the example of B segregation at the Pt Σ3 GB, the segregation energy is given by:

$$E_{seg} = \left(E^{GB}_{Pt+B} - E^{GB}_{Pt}\right) - \left(E^{bulk}_{Pt+B} - E^{bulk}_{Pt}\right)$$

where E^{GB}_{Pt} and E^{GB}_{Pt+B} are the total energies of the Pt GB without and with B, respectively. Similarly, E^{bulk}_{Pt} and E^{bulk}_{Pt+B} represent the total energies of bulk Pt without and with B, respectively. A negative E_{seg} indicates a preference for B to segregate to the GB rather than remain in the bulk.

The mechanism of thermal catalytic hydrogen oxidation reaction modeled in the simulation includes six elementary steps: adsorption of hydrogen followed by dissociation into H*, adsorption of oxygen into O_2*, dissociation of O_2* into two O* atoms, reaction of O* with H* to form OH*, formation of H_2O* from OH* and H*, and desorption of H_2O. Among these, the step involving the reaction between O* and H* to form OH* was identified as the rate-determining step based on the calculated activation barriers.[7]

For the Pt_{201} NP, the activation energy for the dissociation of molecular oxygen (O_2* → 2O*) was calculated to be 0.57 eV. The reaction between O* and H* to form OH*, which is the rate-limiting step in this mechanism, exhibited an activation energy barrier of 1.02 eV. This relatively high barrier suggests a significant kinetic limitation for the dissociative pathway on the surface of the isolated Pt_{201} NP.

In contrast, the GB region of the Pt_{383} Σ3 GB model displayed drastically different energetics. The activation barrier for the O_2* dissociation step was nearly eliminated, with a calculated value of only 0.008 eV. This indicates that the GB structure substantially lowers the energetic requirement for O–O bond cleavage, likely due to the tensile strain and under-coordinated atomic sites inherent to the GB geometry. The subsequent rate-determining step of OH* formation from O* and H* also benefited from a reduced barrier of 0.60 eV. This considerable drop in activation energy compared to the Pt_{201} NP demonstrates the enhanced catalytic efficiency of the GB region in promoting the hydrogen oxidation pathway.

The simulations therefore confirm that while the Pt_{201} NP surface presents a moderate barrier to oxygen activation and a high barrier to the rate-determining hydrogenation of O*, the Pt_{383} Σ3 GB model enables both processes to proceed with significantly lower activation energies. This leads to a higher surface coverage of reactive O* species at the GB, and with sufficient H* coverage available from nearby Pt surfaces, rapid formation of OH* can occur, thereby accelerating the overall hydrogen oxidation reaction.

The thermodynamic equilibrium concentrations of B at Pt Σ3 GBs, expressed in terms of areal concentration, were calculated at 25°C and 400°C using the McLean equation.[8-10]

The form of the McLean isothermal segregation equation we adopted is as follows:

$$C_{GB} = \frac{C_0 \cdot \exp(-\Delta G_{seg}/RT)}{1 + C_0 \cdot (\exp(-\Delta G_{seg}/RT) - 1)}$$

where C_{GB} is the equilibrium B concentration at the GB (atoms/nm²), C_0 is the B concentration in the bulk phase, Δ_{Gseg} is the Gibbs free energy of B segregation at the Pt Σ3 GB (eV), T is the annealing temperature in Kelvin, and R is the universal gas constant (8.314 $J \cdot mol^{-1} \cdot K^{-1}$).

This approach provides a theoretical basis for understanding B segregation levels under equilibrium conditions by quantifying the number of B atoms per unit area at the GB. To enable a direct comparison with experimental data, the measured atomic concentration of B within B-segregated Pt NAs was similarly converted into an areal concentration. This conversion employs the relationship:

$$\text{Areal concentration of boron at Pt GB} = \frac{\text{Number of boron atoms at GB}}{\text{GB area}}$$

where the B atom count at the Pt GB was experimentally determined using APT. For these calculations, the GB region was approximated as a circle with an average diameter of 1.5 nm, characteristic of the B-segregated Pt GBs in the NAs. This method allows for a detailed comparison between theoretical predictions and experimental measurements of B concentration at the Pt GB, providing insights into the thermodynamic behavior of solute segregation in nanoscale GBs under varying thermal conditions.

To determine the impact of B segregation on the stability of the GB, the strengthening energy ($E_{strengthening}$) was calculated as:

$$E_{strengthening} = (E_{GB+B} - E_{GB}) - (E_{FS+B} - E_{FS}),$$

where E_{GB} and E_{GB+B} denote the total energies of the Pt Σ3 GB without and with B segregation, respectively. Similarly, E_{FS} and E_{FS+B} correspond to the total energies of the Pt free surface without and with B segregation, respectively. A negative $E_{strengthening}$ implies that B segregation strengthens the GB, while a positive value suggests a weakening effect.

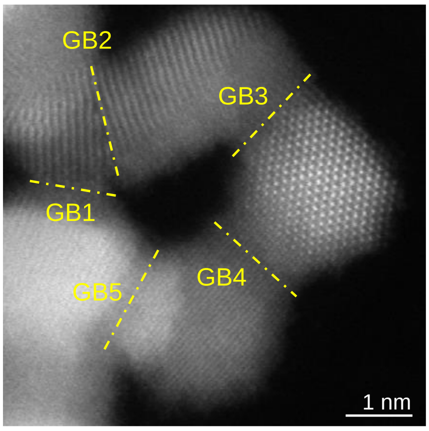

Figure S1. HAADF-STEM image of Pt NAs, with GBs between the NP building blocks highlighted by yellow dashed lines.

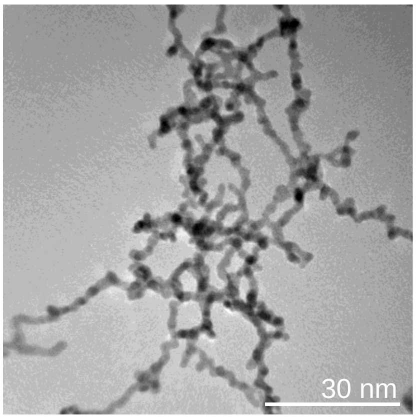

Figure S2. Low-magnification TEM image of Pt NAs.

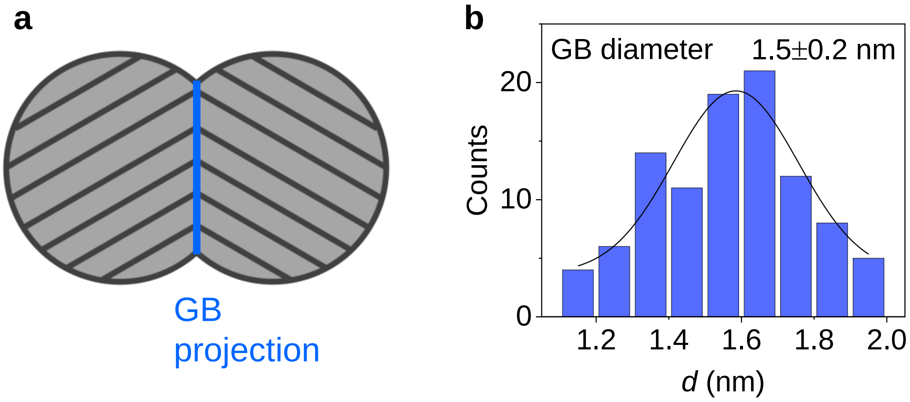

Figure S3. (**a**) The GB plane appears as a linear projection in TEM images. A schematic representation of this projection is shown as a blue line, corresponding to the GB diameter. (**b**) The distribution histogram of GB diameters in B-segregated Pt NAs, obtained by statistically measuring over 100 GB projections from TEM images. The average GB diameter is ~1.5 nm.

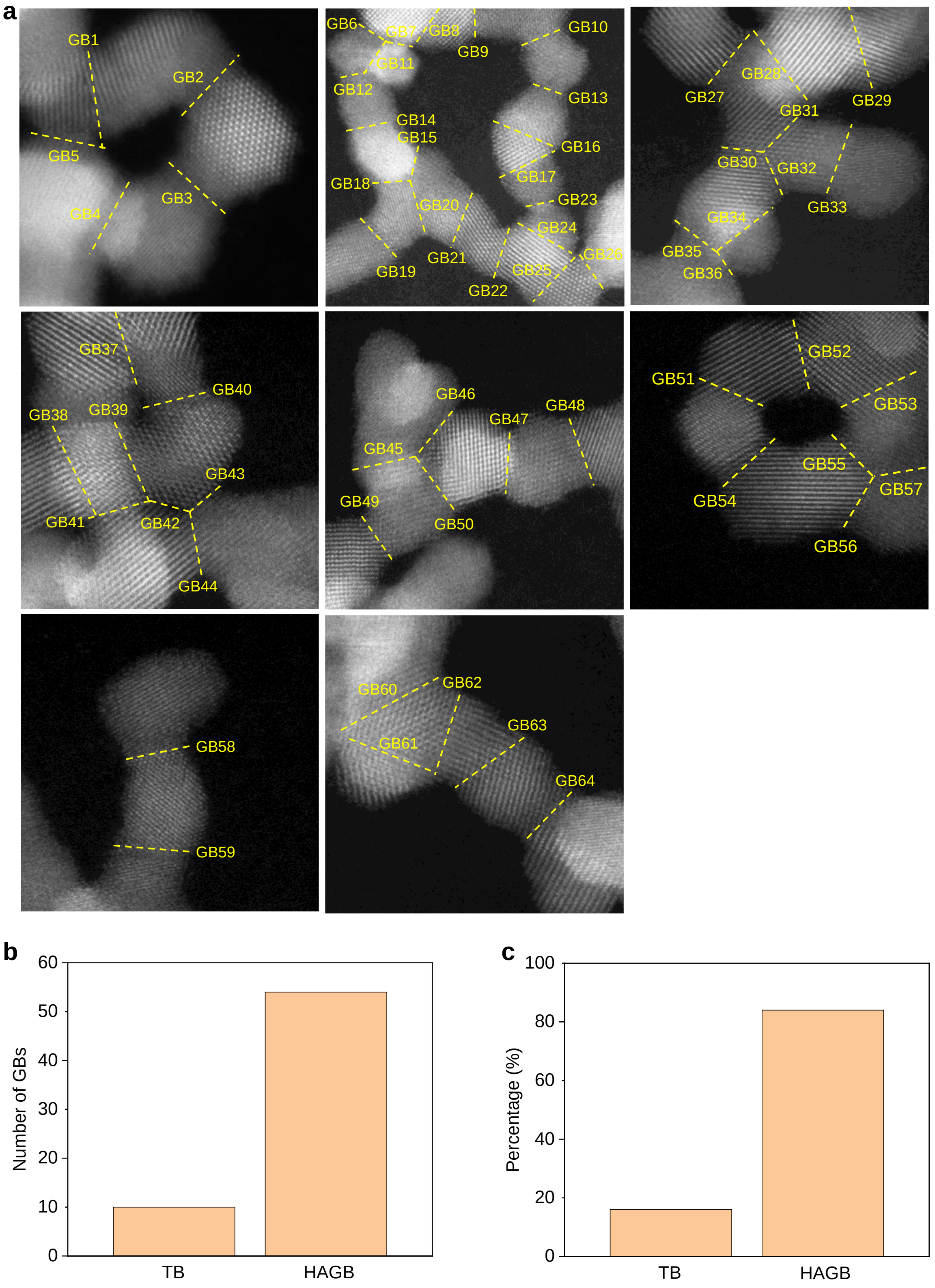

Figure S4. Statistical analysis of GBs in Pt NAs. (**a**) HAADF–STEM images of 64 GBs between Pt NP building blocks within Pt NAs. (**b**) Histogram of the statistical analysis of the 64 GBs identified in panel **a**, showing that 10 are TBs and 54 are HAGBs. (**c**) Relative proportions of TBs and HAGBs derived from the data in panel **b**.

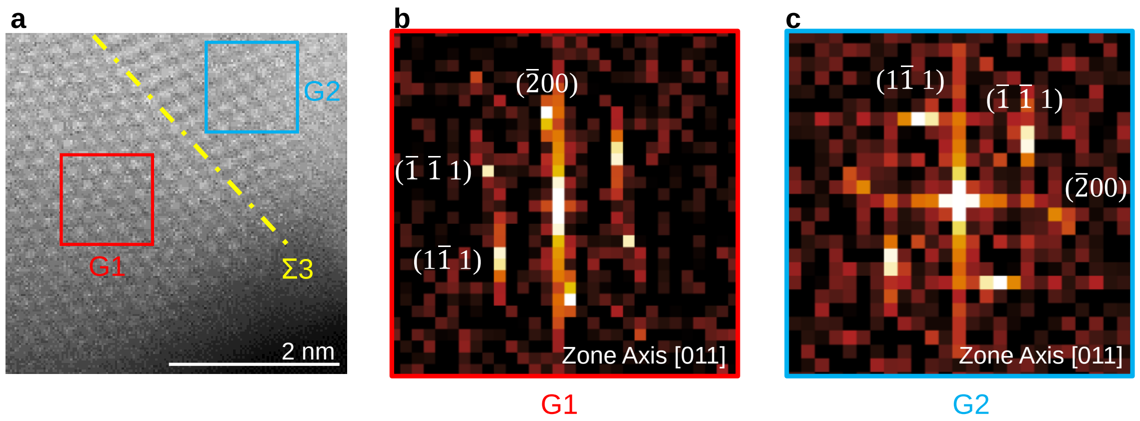

The misorientation angle between G1 and G2 is measured to be 70°, classifying the GB as a Σ3 twin boundary in accordance with CSL theory.

Figure S5. Identification of GB type from HAADF–STEM images. Example illustrating the procedure used to classify GB types, specifically the Σ3 twin boundary (TB) shown in **Figure 2e**. Classification was based on HAADF–STEM imaging combined with crystallographic orientation analysis. According to coincidence site lattice (CSL) theory[11]—where Σ denotes the ratio of total lattice sites to coincidence sites—a GB was identified as a TB when the adjoining grains shared the same zone axis and satisfied the CSL theory for a twinning relationship. In the example (panel **a**, corresponding to **Figure 2e**), the HAADF–STEM image reveals two grains (G1 and G2) on either side of the boundary. Fast Fourier transform patterns from each grain confirm that both have a [011] zone axis, with a measured misorientation of 70°, consistent with a Σ3 TB (panel **b** and **c**).[12] The same analysis method was applied to other GBs: boundaries between grains with the same zone axis were classified as TBs; those with different zone axes and misorientation >15° as high-angle GBs (HAGBs); and those with misorientation <15° as low-angle GBs (LAGBs).

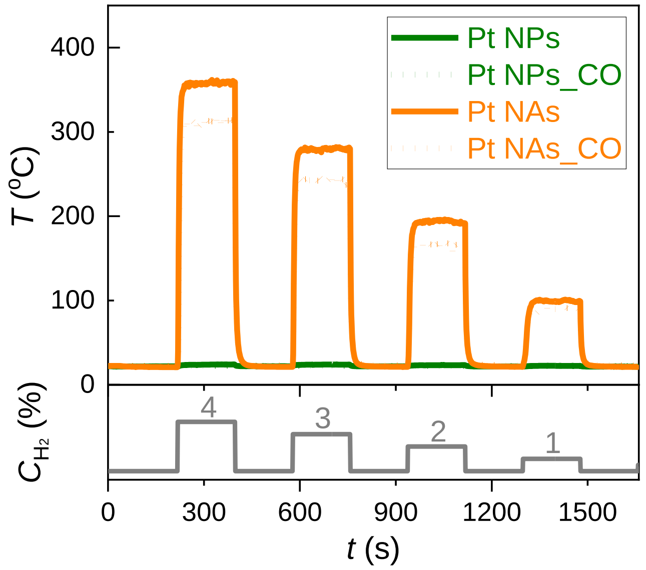

Figure S6. Temperature readout of J-type thermocouples coated with 1 mg of either isolated Pt NPs (olive) or GB-connected Pt NAs, (orange) during stepwise reductions in H_2 concentration from 4% to 1% in air at room temperature (~22 °C), measured both in the absence (solid lines) and presence (dashed lines) of 1000 ppm CO. TCHOR activity ($H_2 + ½O_2 \rightarrow H_2O$) was evaluated by the exothermic temperature rise induced upon H_2 exposure. Prior to H_2 introduction, thermocouples stabilized at room temperature. Upon exposure to 4% H_2, Pt NAs generated a rapid temperature increase to ~400 °C, while Pt NPs exhibited only a slight rise, indicating limited activity. As H_2 concentration decreased, the temperature rise diminished accordingly, demonstrating a clear H_2 concentration-dependent catalytic response. The greater temperature change in Pt NAs reflects a significantly lowered activation barrier at GB sites, enabling efficient ambient-temperature TCHOR. In contrast, isolated Pt NPs lacked sufficient activity under identical conditions due to high kinetic barriers. In the presence of 1000 ppm CO, Pt NAs retained substantial activity, whereas Pt NPs showed marked deactivation, confirming the superior CO tolerance of GB-connected structures.

To quantitatively compare the TCHOR catalytic activity of Pt NPs and Pt NAs, the mass activity was calculated based on the temperature increase upon exposure to H_2. Specifically, mass activity per mg of Pt is defined as the temperature rise normalized to the baseline temperature and Pt loading: Mass activity (per mg Pt) = (T_H₂ – T_initial) / (T_initial × 1 mg Pt). Take Pt NAs towards 4% H_2 as an example, the temperature increases from ~22 °C to ~400 °C during 4% H_2 exposure, yielding: Mass activity (per mg Pt) = (400 °C – 22 °C) / (22 °C × 1 mg Pt). This mass activity (per mg Pt) enables a direct and normalized comparison of TCHOR catalytic activity between Pt NPs and Pt NAs under identical conditions.

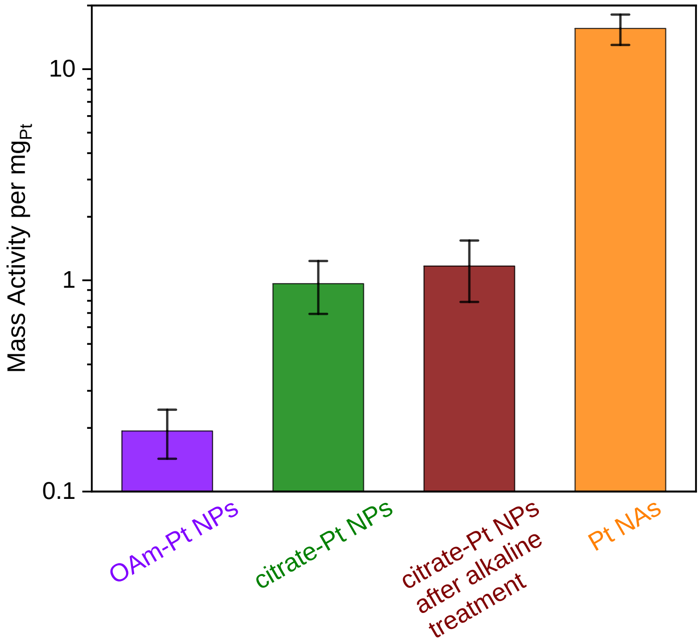

Figure S7. Mass activity for TCHOR over OAm-capped Pt NPs, citrate-capped Pt NPs, citrate-capped Pt NPs after NaOH treatment (ligand-free), and Pt NAs. OAm-capped Pt NPs exhibit very low activity—nearly an order of magnitude lower than citrate-capped Pt NPs—due to the long-chain OAm strongly binding to surface Pt atoms and blocking active sites. Ligand-free Pt NPs show only a slight activity increase relative to citrate-capped Pt NPs, as citrate is a short-chain ligand that binds weakly to Pt and minimally hinders access of small reactants such as H_2 and O_2. In contrast, Pt NAs display over an order of magnitude higher activity than ligand-free Pt NPs, highlighting GBs as highly active catalytic sites for TCHOR.

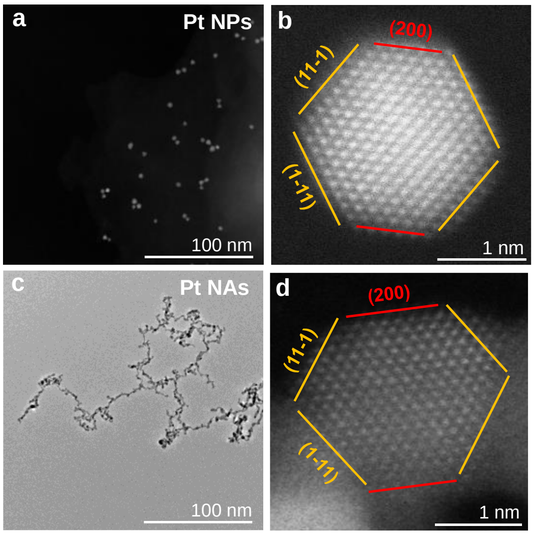

Figure S8. (**a**) High-resolution and (**b**) atomic-resolution HAADF-STEM images of Pt NPs before annealing, showing uniform grain size and (111) and (200) surface facets. (**c**) High-resolution TEM and (**d**) atomic-resolution HAADF-STEM images of B-free Pt NAs prior to annealing, also exhibiting dominant (111) and (200) facets.

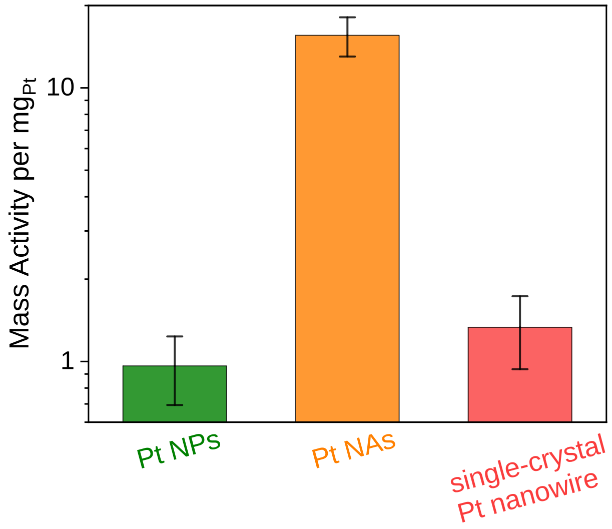

Figure S9. Effect of geometry on TCHOR activity. (**a**) TEM image of single-crystal Pt nanowires (almost GB-free) synthesized using a reported method.[13] These single-crystal Pt samples exhibit a porous, nanowire-shaped geometry similar to that of Pt NAs, but differ in their GB content—single-crystal Pt nanowires are nearly GB-free, whereas Pt NAs are GB-rich. (**b**) Comparison of catalytic mass activity for TCHOR over Pt NPs, Pt NAs, and single-crystal Pt nanowires. Single-crystal Pt nanowires show slightly higher activity than Pt NPs, with the two being comparable overall. In contrast, Pt NAs exhibit over an order of magnitude higher activity than both, indicating GBs as highly active catalytic sites for TCHOR.

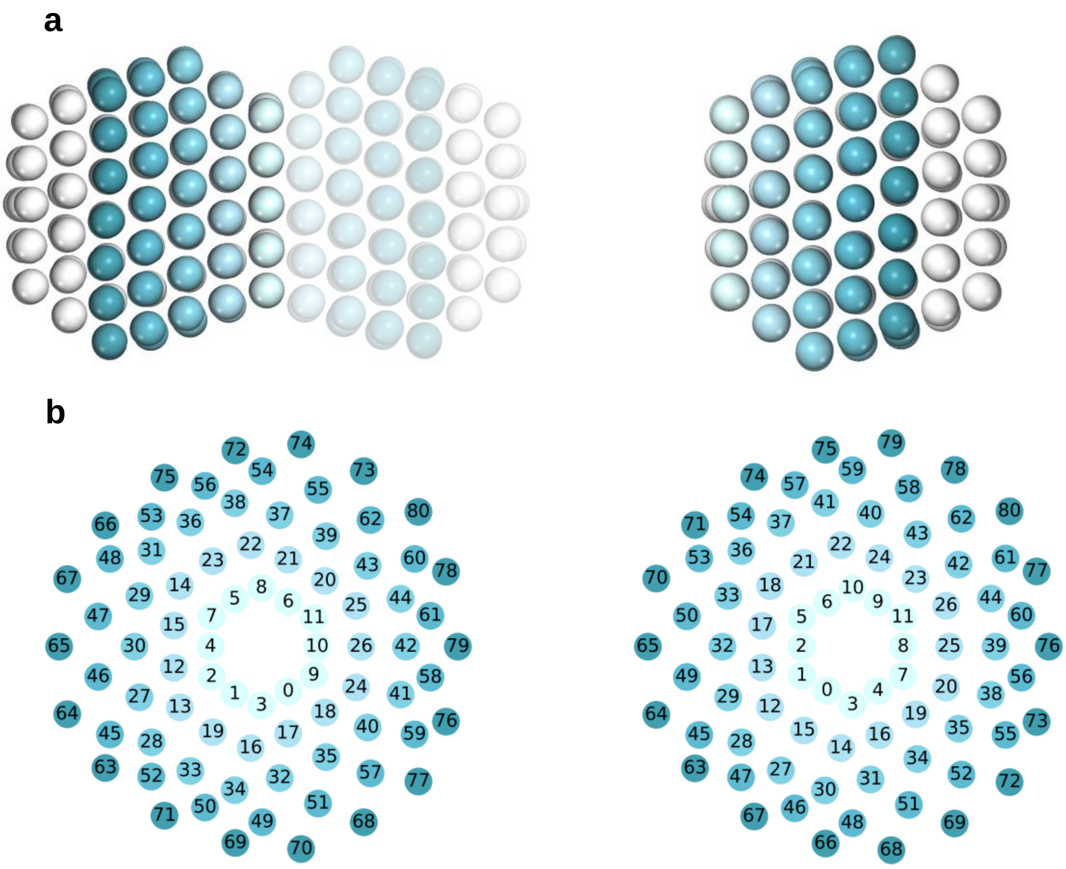

Figure S10. Structural and electronic characterization of a Σ3 GB formed by the connection of two NPs with a diameter of approximately 2 nm. DFT simulations were used to compare the electronic structure of 80 surface Pt atoms from five atomic layers near the GB with the corresponding surface atoms from an isolated NP of same size. (**a**) Side view of the Pt_{383} Σ3 [110] (111) GB model (left) and the Pt_{201} NP model (right), with surface atoms near the GB highlighted in blue. The shading intensity indicates the proximity to the GB, where lighter blue represents atoms closer to the GB, and darker blue indicates atoms farther from the GB. (**b**) Radial indexing of surface Pt atoms from the DFT simulation, projected onto concentric circles for visualization. Atoms closer to the GB are positioned near the center of the concentric circles, while atoms farther away are located towards the outermost circle, corresponding to the surface layer furthest from the GB.

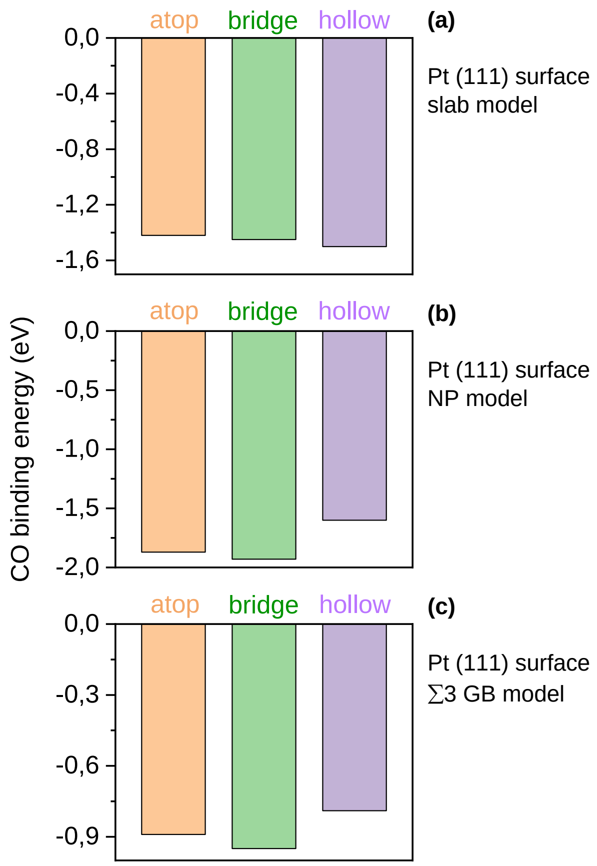

Figure S11. Comparative analysis of the binding energy of a CO molecule on different adsorption sites–atop, bridge, and hollow–on the Pt (111) surface for three distinct models: slab, NP, and Σ3 GB models. The variation in CO binding energies across these adsorption sites provides insights into the electronic environment and anti-CO poisoning effect of Pt in different structural configurations.

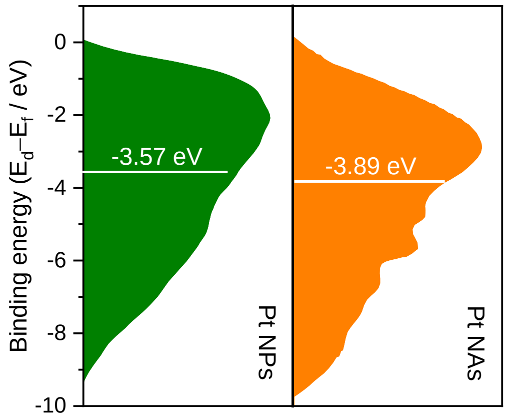

Figure S12. The surface valence band photoemission spectra of Pt NPs and Pt NAs. The Pt NAs display a noticeable downward shift in the valence band center relative to Pt NPs, indicating a reduced electronic density of states near the Fermi level. This shift is attributed to the high density of GBs in Pt NAs, which modulate the local electronic structure. The electronic modification is consistent with the observed improvement in CO-tolerance.

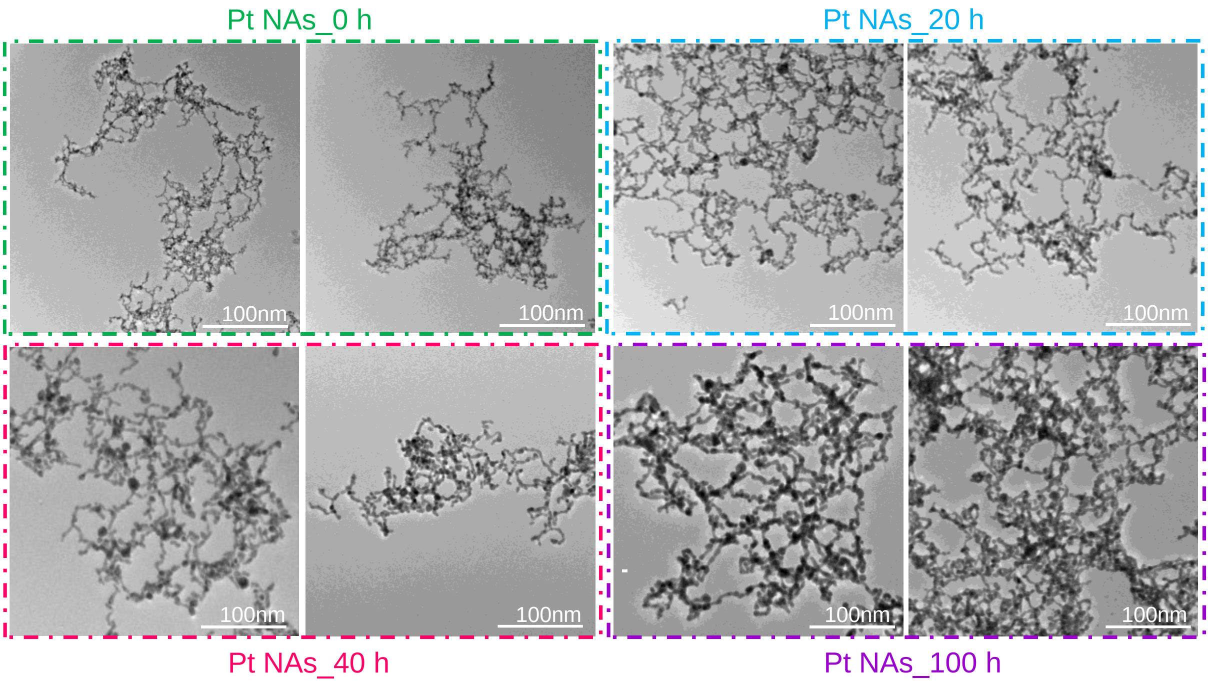

Figure S13. HRTEM images collected from multiple regions of B-free Pt NAs before annealing, and after vacuum annealing at 400 °C for 20 h, 40 h, and 100 h, respectively. The images illustrate the progressive grain coarsening and structural evolution induced by thermal treatment over time.

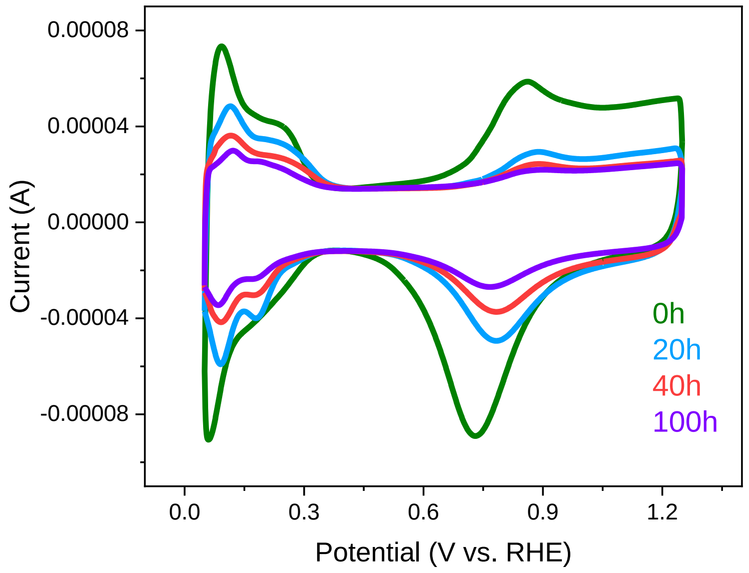

Figure S14. Cyclic voltammograms of boron-free Pt NAs as a function of vacuum annealing time at 400 °C, recorded in 0.1 M $HClO_4$ over a potential range of 0.05 V to 1.25 V at a scan rate of 50 mV/s.

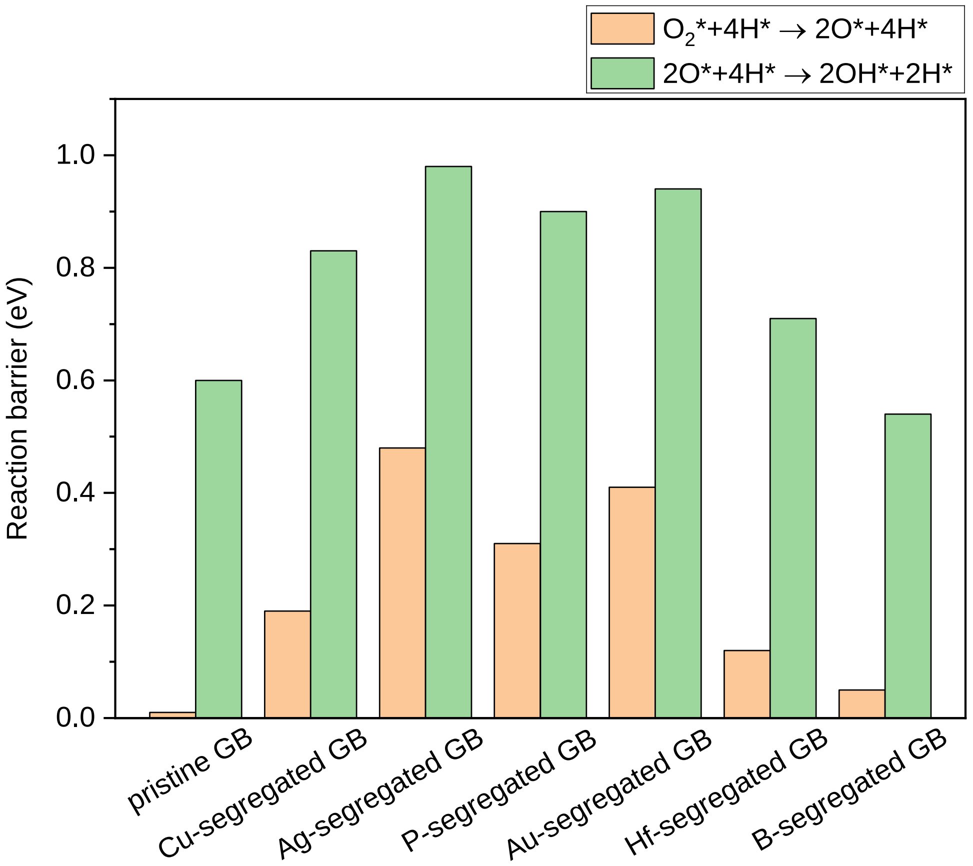

Figure S15. Effect of segregating elements on the reaction barriers of TCHOR. Reaction energy barriers for the key steps in the TCHOR pathway—O_2* + 4H* → 2O* + 4H* and 2O* + 4H* → 2OH* + 2H*—are shown for a pristine Pt Σ3 GB and for GBs segregated with strongly segregating elements (Cu, Ag, P, Au, Hf, and B). B segregation results in barriers comparable to those of the pristine GB, indicating minimal impact on TCHOR activity. In contrast, segregation of Cu, Ag, P, Au, and Hf substantially increases the barriers, suggesting a detrimental effect on catalytic performance.

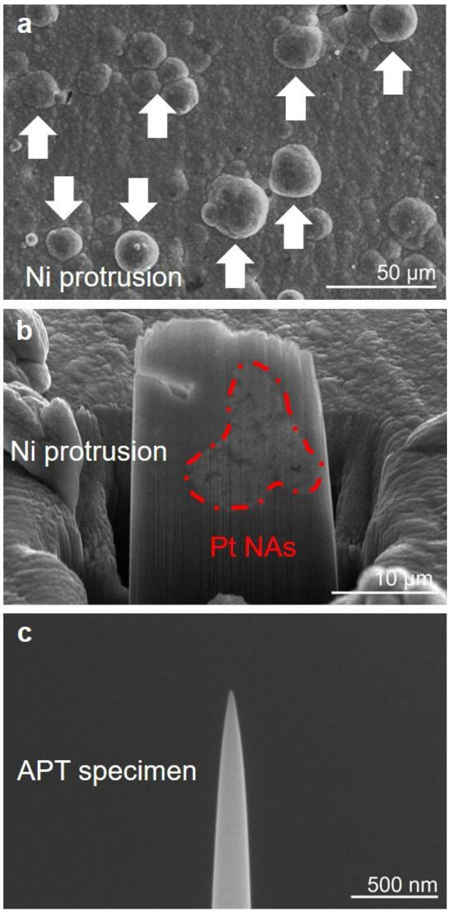

Figure S16. (**a**) Cross-sectional FIB-SEM image of the protrusion. (**b**) Cross-sectional ion-beam image of the protrusion, showing Pt NAs (outlined by a red dashed line) embedded in the Ni matrix. (**c**) A sharpened specimen of Pt NAs embedded in the Ni matrix prepared for APT measurement.

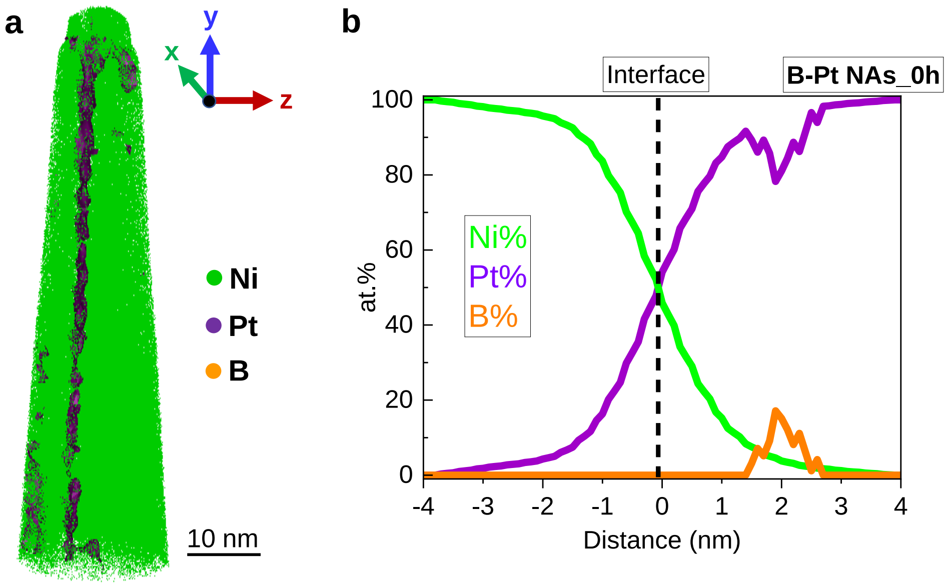

Figure S17. Atomic-scale analysis of unannealed B-Pt NAs within Ni matrix. (**a**) Three-dimensional atom map of unannealed B-Pt NAs, embedded within Ni matrix, depicted by Pt iso-surfaces (indicating regions with >50 atomic % Pt), illustrating the spatial distribution of Pt and boron. (**b**) Proximity histogram of Ni, Pt, and B concentrations across the Ni matrix/unannealed B-Pt NAs interface, demonstrating that boron atoms predominantly reside within the interior of the unannealed B-Pt NAs rather than accumulating on the surface.

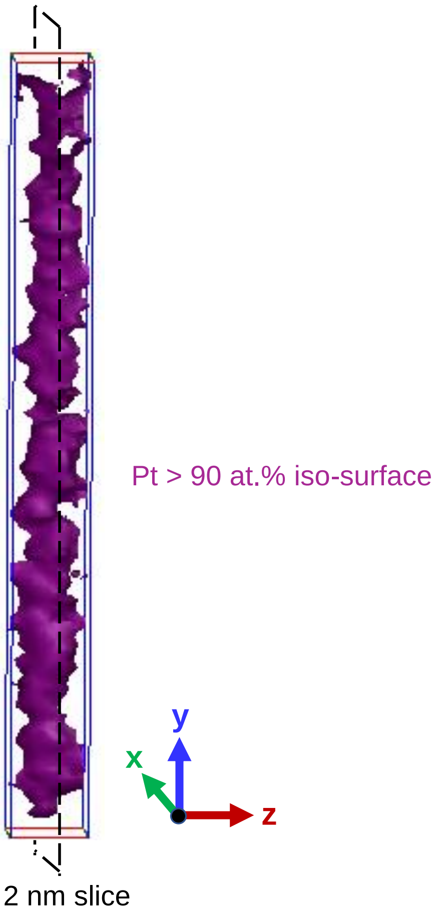

Figure S18. 3D atom map of Pt NAs (Pt>90 at.% iso-surface).

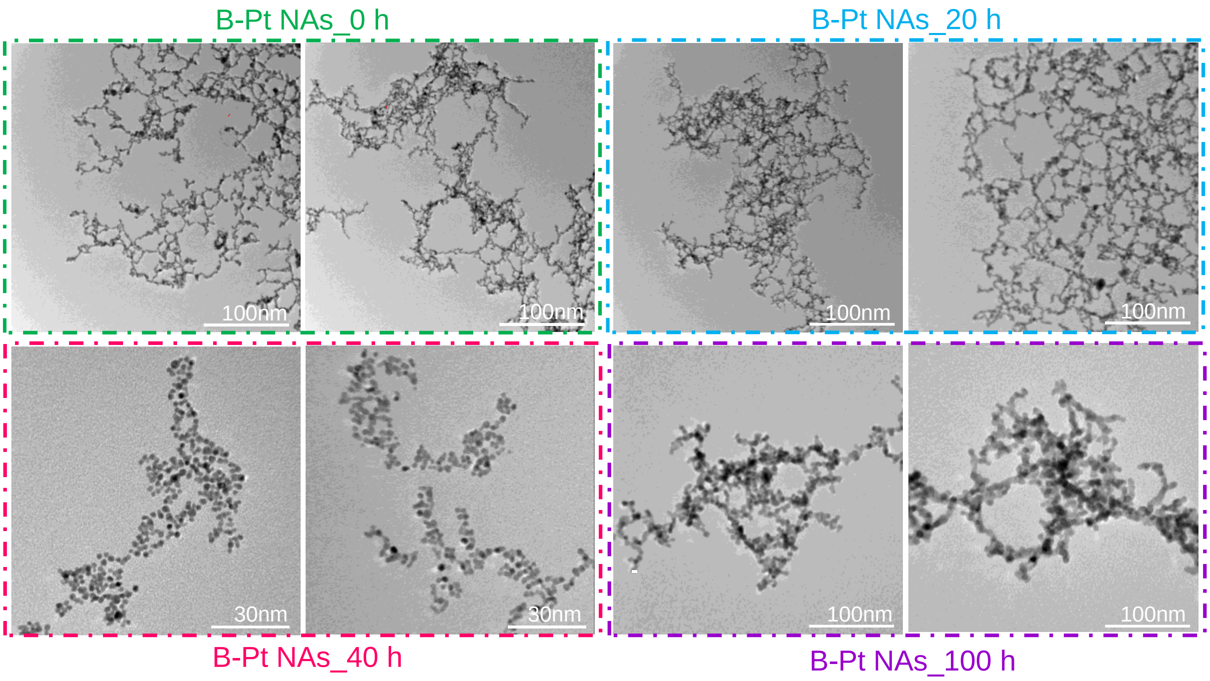

Figure S19. HRTEM images collected from multiple regions of B-Pt NAs before annealing, and after vacuum annealing at 400 °C for 20 h, 40 h, and 100 h, respectively. To clearly visualize particle separation in B3, images with higher magnification were selected. For other samples, slightly lower magnification images were used to better display the overall structural features. These images collectively reveal the grain coarsening and structural evolution of B-Pt NAs during extended thermal treatment.

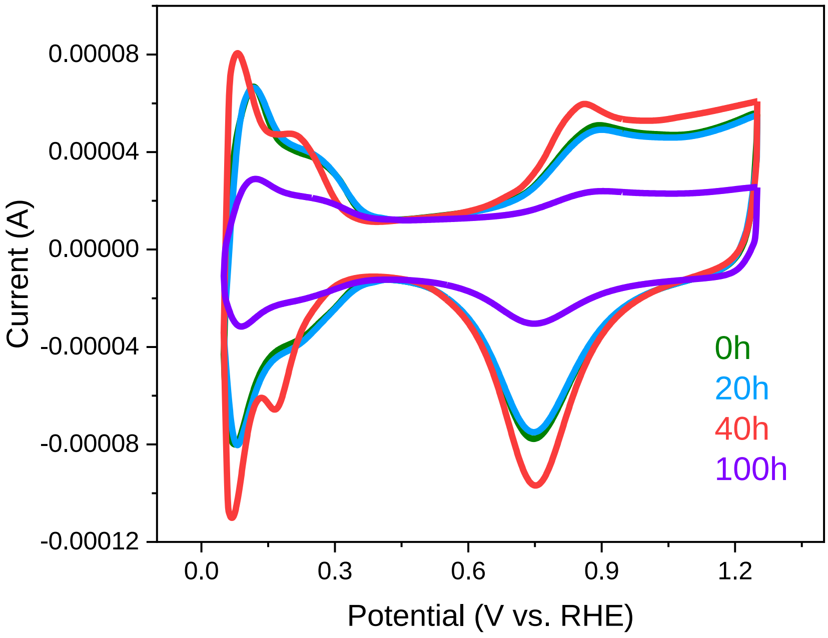

Figure S20. Cyclic voltammograms of B-Pt NAs as a function of vacuum annealing time at 400 °C, recorded in 0.1 M $HClO_4$ over a potential range of 0.05 V to 1.25 V at a scan rate of 50 mV/s.

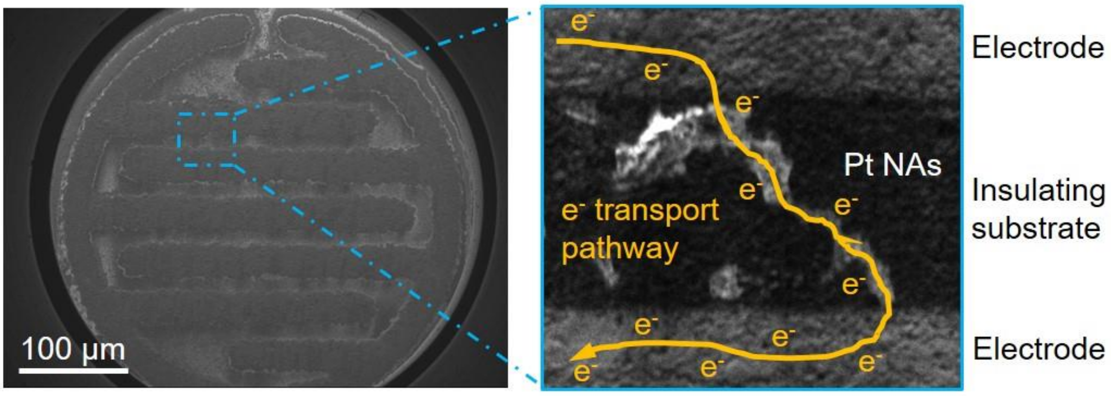

Figure S21. (**a**) Low-magnification field-emission scanning electron microscopy (FE-SEM) image of loosely dispersed Pt NAs by spin-coating. (**b**) High-magnification FE-SEM image of the marked area, illustrating the connection between adjacent electrodes by single Pt NAs on the insulating substrate.

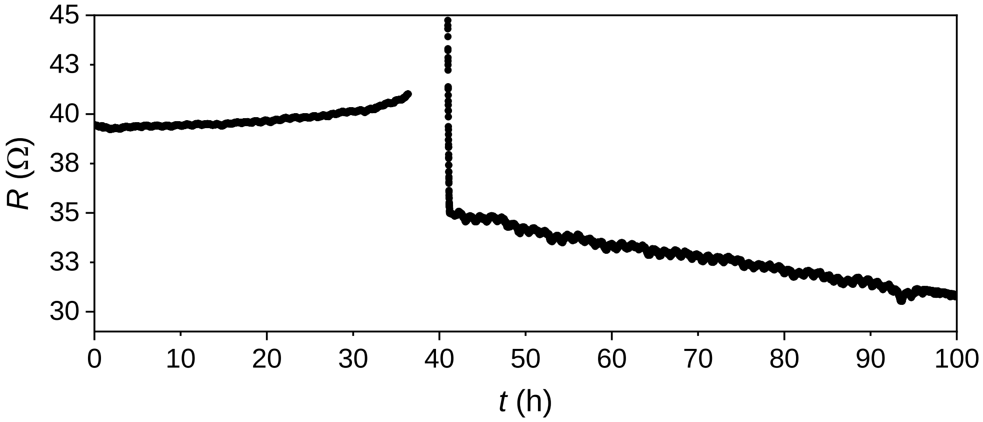

Figure S22. Electrical resistance evolution of B-Pt NAs during vacuum annealing at 400 °C over 100 hours, capturing dynamic structural degradation through real-time conductivity monitoring.

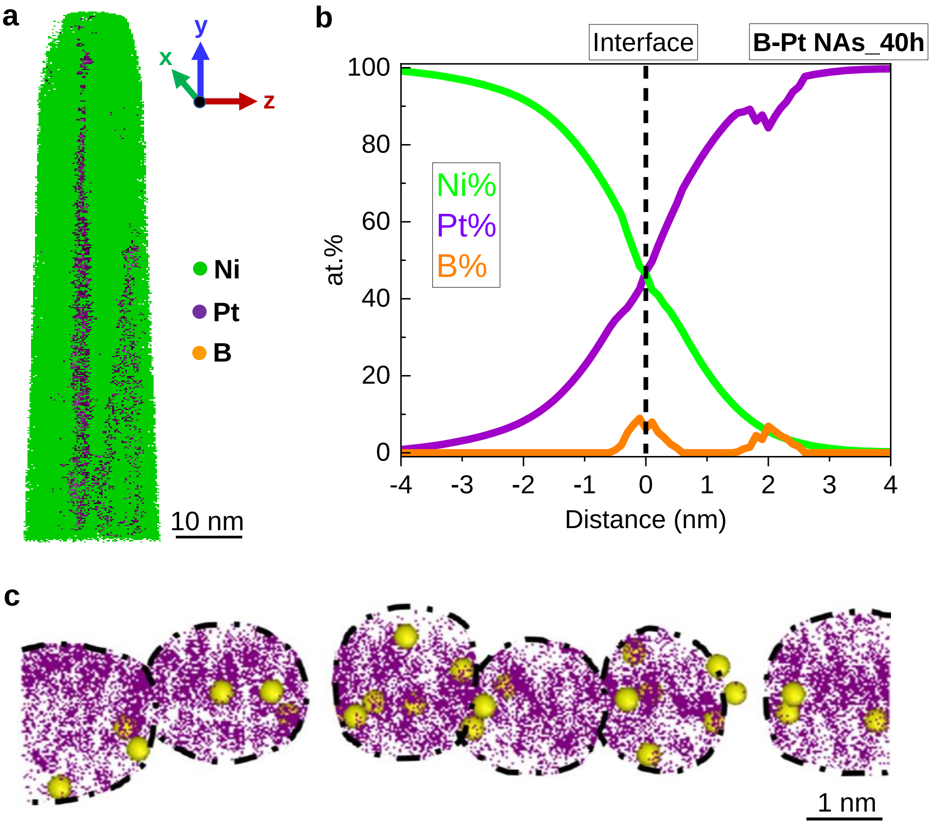

Figure S23. Atomic-scale characterization of B-Pt NAs after annealing of 40 hours (denoted as B-Pt NAs_40 h), revealing boron redistribution and GB decohesion. (**a**) Three-dimensional atom map of B-Pt NAs_40 h embedded in Ni matrix, marked by Pt iso-surfaces (>50 atomic % Pt), illustrating the nanoscale distribution of Pt and boron. (**b**) Proximity histogram of Ni, Pt, and B across the Ni matrix/B-Pt NAs_40 h interface, indicating the presence of boron at both the NA interior and surface, in contrast to unannealed B-Pt NAs where boron resides primarily inside. This redistribution suggests boron migration after annealing at 400°C for 40 hours. (**c**) A 2-nm thin tomographic slice from the 3D atom map (iso-composition surface >90 atomic % Pt), showing GB features delineated by a dotted black line, with yellow spheres representing B atoms. Notably, GB decohesion is observed as gaps between some NP building blocks, with boron accumulating at decohered GB surfaces, suggesting boron accumulation may drive GB decohesion.

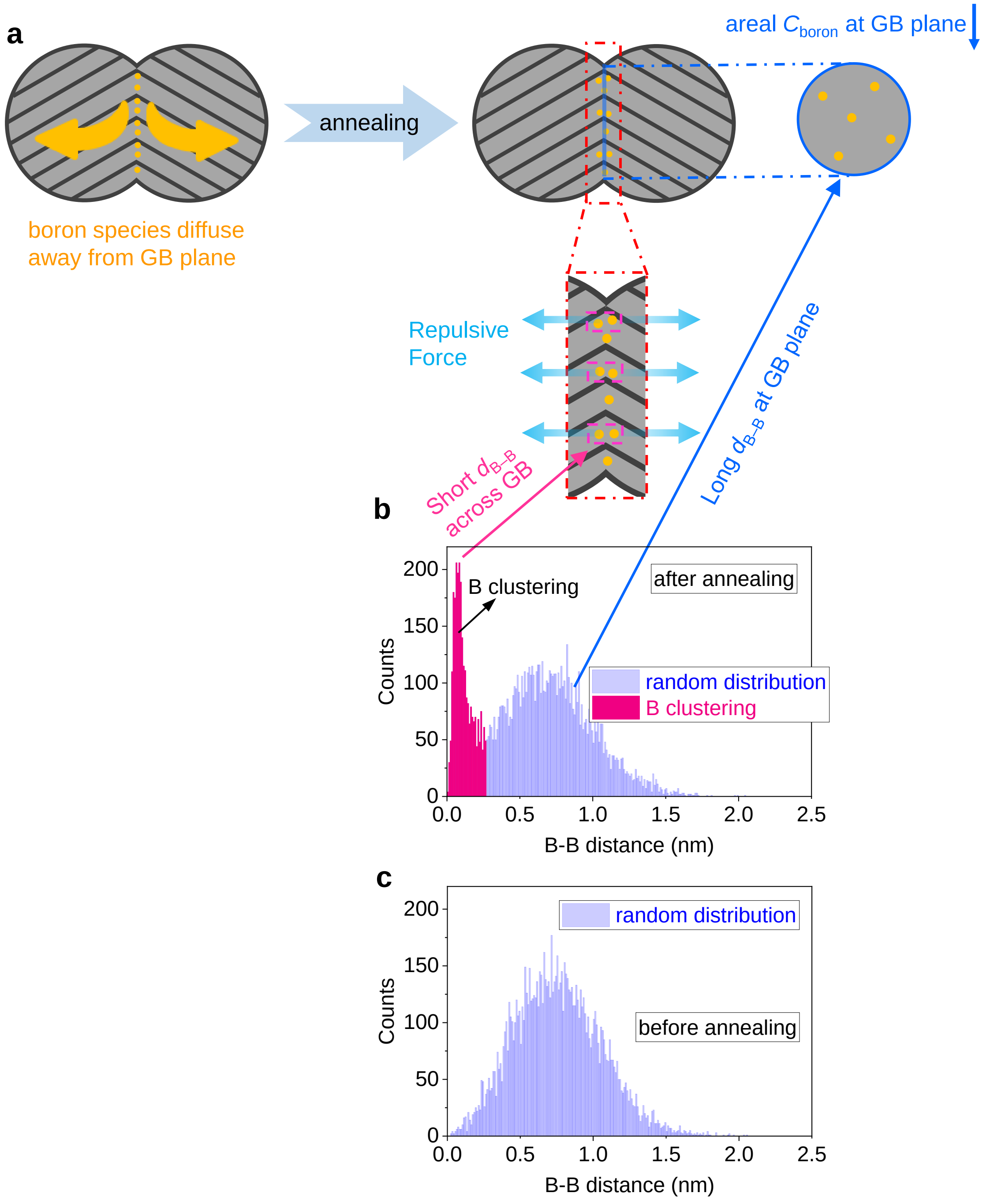

Figure S24. (**a**) Schematic illustration of the formation mechanism of B–B clusters near GBs. Before annealing, B atoms are primarily segregated within the GB plane and exhibit a random distribution. Upon thermal treatment, when the temperature is sufficiently high to overcome the diffusion barrier, a portion of the B atoms diffuse from the GB plane into the adjacent grain interiors, establishing a concentration gradient perpendicular to the GB. However, this diffusion is spatially constrained by dislocation networks, local strain fields, and interfacial discontinuities. As a result, B atoms accumulate within a few atomic layers near the GB. Under these confined conditions, local clustering of B atoms occurs, forming nonequilibrium B-rich regions with short B–B distances. These clusters increase local strain energy and introduce strong repulsive interactions across the GB, disrupting Pt–Pt bonding and triggering GB decohesion. Meanwhile, the remaining B atoms that stay within the GB plane retain a random distribution and long B–B spacing. (**b**) Experimental evidence of this mechanism is provided by the B–Pt NAs sample, after vacuum annealing at 400 °C for 40 h. This sample exhibits clear signs of GB decohesion and nanoparticle separation. Nearest-neighbor distribution analysis from APT data reveals two distinct B–B distance populations: a broad distribution above 0.5 nm (shaded blue region) and a pronounced peak below 0.3 nm (shaded pink region), indicating local B clustering. DFT calculations show that B–B distances below 0.4 nm significantly weaken GB cohesion, rendering the boundary thermodynamically unstable. No such GB decohesion or particle separation is observed in B-free Pt NAs, confirming that the formation of B clusters under thermal treatment is the primary driving force behind GB destabilization and subsequent nanoparticle disconnection. (**c**) Nearest-neighbor distribution analysis of B–Pt NAs prior to annealing shows a broad B–B distance distribution, indicative of a random and unclustered arrangement of B atoms near the GBs.

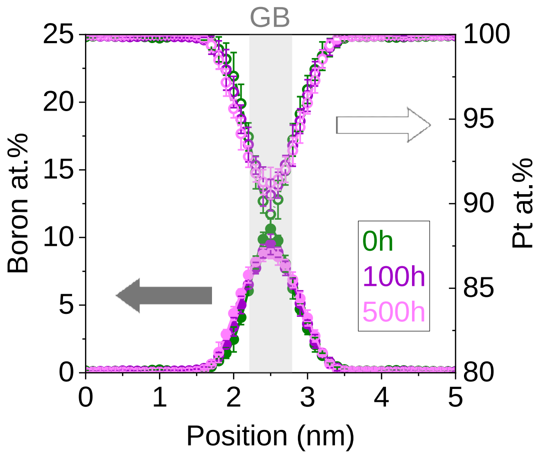

Figure S25. (**a**) Statistical analysis of B distribution near GBs for BL-Pt NAs after annealing for 0, 40, and 100 hours, derived from one-dimensional compositional profiles across at least 20 GBs within a cylindrical region of interest ($\Phi 1.5\times 10$ nm^3) perpendicular to the GB. (**b**) Areal concentration of B at GBs in BL-Pt NAs as a function of annealing time at 400°C in vacuum, calculated using APT.

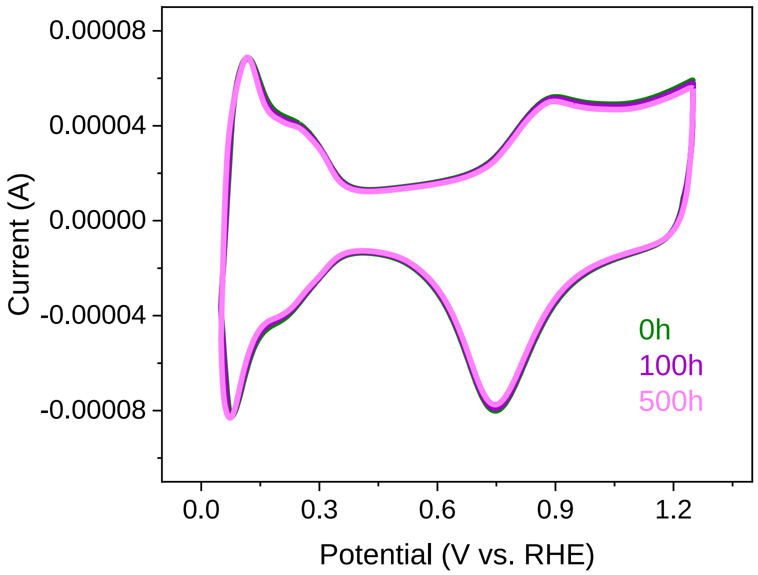

Figure S26. Cyclic voltammograms of BL-Pt NAs as a function of vacuum annealing time at 400 °C, recorded in 0.1 M $HClO_4$ over a potential range of 0.05 V to 1.25 V at a scan rate of 50 mV/s.

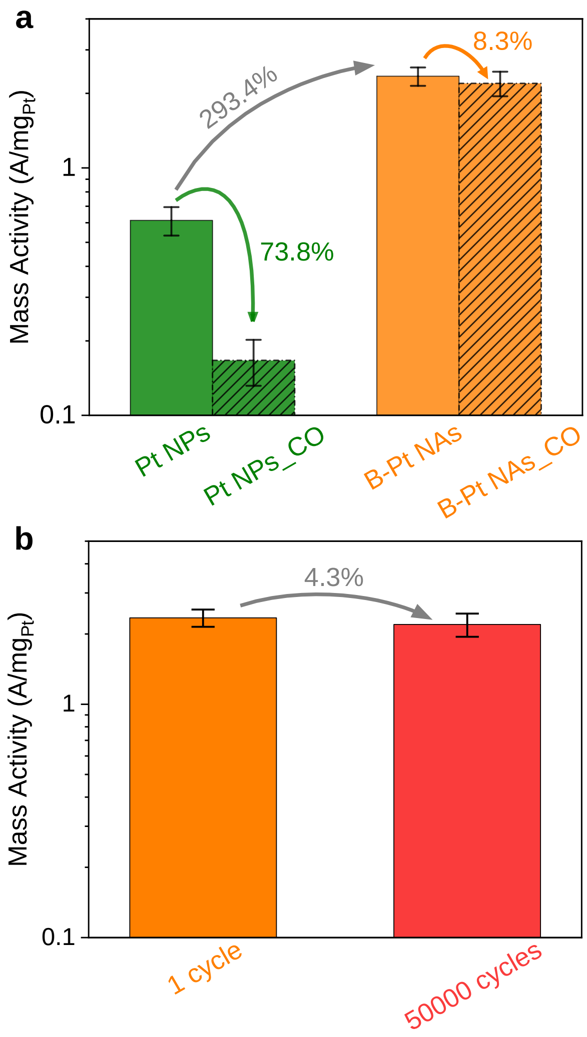

Figure S27. (**a**) Mass activity for the methanol oxidation reaction (MOR) of Pt NPs and B-Pt NAs with identical Pt loading, measured in the presence and absence of 1000 ppm CO. The MOR activity of B-Pt NAs is over two orders of magnitude higher than that of Pt NPs, indicating that GBs serve as highly active sites for MOR. Furthermore, B-Pt NAs exhibit significantly less activity degradation in the presence of CO, suggesting superior resistance to CO poisoning compared to Pt NPs. (**b**) Mass activity of B-Pt NAs during the first cycle and after 50000 cycles. The MOR activity decreases by 4.3% after 50000 cycles, demonstrating excellent structural stability of the B-Pt NAs. Error bars indicate the standard deviation from measurements taken at least three times.

Table S1. Comparison of acidic MOR performance of BL-Pt NAs and state-of-art Pt-based nanocatalysts from recent published works.

Catalyst	Specific activity (mA/cm^2)	Mass activity (A/mg$_{Pt}$)	References
Pt/Nb nanocrystal	NA	2.7	ACS Catal. 2025, 15, 4350–4358
PtNiCu nanocrystal	3.3	1.5	Cell, 2025, in press https://doi.org/10.1016/j.matt.2025.102096
Pt nanowire	8.7	2.5	Adv. Sci. 2024, 11, 2309813
Pt nanocube	NA	2.94	Nature 2021, 598, 76–81
Pt/Ni(OH)$_2$/rGO	NA	1.24	Nat. Catal. 2021, 4, 830–839
PtNiRh nanowire	2.49	1.72	Adv. Mater. 2019, 31, 1805833
PtCoAu NPs	NA	1.49	Angew. Chem. Int. Ed. 2019, 131, 11651–11657
Pt$_3$Ga film	7.20	1.09	J. Am. Chem. Soc. 2018, 140, 2773–2776
PtAg nanotube	6.63	2.08	Chem. Mater. 2018, 30, 7744–7751
PtPdRuFe nanotube	2.96	1.26	J. Am. Chem. Soc. 2017, 139, 5890–5895
PtCu nanotube	6.09	2.25	Energy Environ. Sci. 2017, 10, 1751–1756
PtRu NPs	10.98	1.7	Adv. Energy Mater. 2017, 7, 1601593
PtCu nanofoam	7.5	3.3	ACS Nano 2017, 11, 11946–11953
PtCo nanowire	1.95	1.02	Nat. Commun. 2016, 7, 11850
PtNi nanofoam	1.90	1.04	Nano Lett. 2016, 16, 2762–2767
PtPb nanoparticle	7.95	1.53	Electrochim. Acta 2012, 63, 346–353
PtCu nanocube	4.7	NA	Angew. Chem. Int. Ed. 2009, 48, 4217–4221
PtAu nanoparticle	3.4	NA	J. Am. Chem. Soc. 2013, 135, 7985–7991
PtRu nanoparticle	3	1.43	Electrochim. Acta 2012, 63, 346–353
PtPb nanoplate	2.7	1.5	Science 2016, 354, 1410–1414
PtPb$_{0.27}$ nanowire	2.41	1.21	Chem. Mater. 2016, 28, 4447–4452
Pt$_7$FeRu$_2$ nanowire	2.27	NA	Energy Environ. Sci. 2015, 8, 350–363
Pt$_3$Cu nanoicosahedra	2.14	0.74	ACS Nano 2015, 9, 7634–7640
PtPd nanocube	2	NA	J. Am. Chem. Soc. 2011, 133, 3816–3819
Pt-Ni concave-nanocube	1.86	0.17	Angew. Chem. Int. Ed. 2014, 53, 12522–12527
Pt-Ni hexoctahedra	1.71	0.14	Angew. Chem. Int. Ed. 2014, 53, 12522–12527
Pt$_3$Cu nanoctahedra	1.63	0.52	ACS Nano 2015, 9, 7634–7640
PtNi concave nanoctahedra	1.55	0.44	Angew. Chem. Int. Ed. 2012, 51, 12524–12528
Pt$_3$Co nanocube	1.5	NA	Angew. Chem. Int. Ed. 2010, 49, 6848–6851

Pt-Si alloy	1.5	NA	J. Phys. Chem. C 2015, 119, 8023–8031
Pt-Ni nanocube	1.40	0.11	Angew. Chem. Int. Ed. 2014, 53, 12522–12527
$Pt_{93}Ru_7$ alloy	1.2	NA	J. Phys. Chem. 1993, 97, 12020–12029
PtZn nanoparticle	0.92	NA	ACS Nano 2012, 6, 5642–5647
Pt_3V nanoparticle	0.38	0.20	J. Am. Chem. Soc. 2014, 136, 10206–10209
Pt_3Ti nanoparticle	0.31	0.15	J. Am. Chem. Soc. 2014, 136, 10206–10209
PtPb/CNTs	NA	1.57	Int. J. Hydrogen Energy 2012, 37, 1263–1271
PtPb nanoparticle	NA	0.78	J. Power Sources 2008, 184, 16–22
PtPb nanorod	NA	0.70	J. Am. Chem. Soc. 2007, 129, 8684–8685
$Pt_{38}Fe_{28}Pd_{34}$ nanowire	NA	0.49	J. Am. Chem. Soc. 2011, 133, 15354–15357
Pt-Ru-TaO_x-PbO_x	NA	0.28	J. Electroanal. Chem. 2012, 668, 13–25
PtPb alloy	NA	0.14	Electrochem. 2012, 80, 132–138
**BL-Pt NAs**	**3.31**	**2.32**	**This work**

Supplementary References

1 Kresse, G. & Hafner, J. Ab initio molecular dynamics for liquid metals. *Physical review B* **47**, 558 (1993).

2 Kresse, G. & Furthmüller, J. Efficiency of ab-initio total energy calculations for metals and semiconductors using a plane-wave basis set. *Computational materials science* **6**, 15-50 (1996).

3 Blöchl, P. E. Projector augmented-wave method. *Physical review B* **50**, 17953 (1994).

4 Geng, X. *et al.* Concave Grain Boundaries Stabilized by Boron Segregation for Efficient and Durable Oxygen Reduction. *Advanced Materials*, 2404839 (2024).

5 Perdew, J. P., Burke, K. & Ernzerhof, M. Generalized gradient approximation made simple. *Physical review letters* **77**, 3865 (1996).

6 Hammer, B., Hansen, L. B. & Nørskov, J. K. Improved adsorption energetics within density-functional theory using revised Perdew-Burke-Ernzerhof functionals. *Physical review B* **59**, 7413 (1999).

7 Geng, X. *et al.* Grain‐boundary‐rich noble metal nanoparticle assemblies: synthesis, characterization, and reactivity. *Advanced Functional Materials* **32**, 2204169 (2022).

8 Wagih, M. & Schuh, C. A. Grain boundary segregation beyond the dilute limit: Separating the two contributions of site spectrality and solute interactions. *Acta Materialia* **199**, 63-72 (2020).

9 Westbrook, J. Segregation at grain boundaries. *Metallurgical Reviews* **9**, 415-471 (1964).

10 McLean, D. & Maradudin, A. (American Institute of Physics, 1958).

11 Grimmer, H., Bollmann, W. & Warrington, D. Coincidence-site lattices and complete pattern-shift in cubic crystals. *Foundations of Crystallography* **30**, 197-207 (1974).

12 Song, M. *et al.* Oriented attachment induces fivefold twins by forming and decomposing high-energy grain boundaries. *Science* **367**, 40-45 (2020).

13 Xia, B. Y., Wu, H. B., Yan, Y., Lou, X. W. & Wang, X. Ultrathin and ultralong single-crystal platinum nanowire assemblies with highly stable electrocatalytic activity. *Journal of the American chemical Society* **135**, 9480-9485 (2013).
